# Quantification of Human Oral and Fecal *Streptococcus parasanguinis* by Use of Quantitative Real-Time PCR Targeting the *groEL* Gene

**DOI:** 10.3389/fmicb.2019.02910

**Published:** 2019-12-20

**Authors:** Qiurong Chen, Guojun Wu, Hui Chen, Hui Li, Shuo Li, Chenhong Zhang, Xiaoyan Pang, Linghua Wang, Liping Zhao, Jian Shen

**Affiliations:** ^1^Key Laboratory of Systems Biomedicine (Ministry of Education), Shanghai Center for Systems Biomedicine, Shanghai Jiao Tong University, Shanghai, China; ^2^State Key Laboratory of Microbial Metabolism, School of Life Sciences and Biotechnology, Shanghai Jiao Tong University, Shanghai, China

**Keywords:** *Streptococcus parasanguinis*, quantitative PCR, *groEL* gene, feces, saliva

## Abstract

Two pairs of species-specific PCR primers targeting the housekeeping *groEL* gene, Spa146f-Spa525r and Spa93f-Spa525r, were designed to quantify human oral and fecal *Streptococcus parasanguinis*. Blast analysis against reference sequences of NCBI nucleotide collection database and the Chaperonin Sequence Database showed the forward primers Spa146f and Spa93f 100% matched only with *S. parasanguinis*, and the *in silico* Simulated PCR algorithm showed both primer pairs hit only *S. parasanguinis groEL* gene in Chaperonin Sequence Database. The two primer pairs were respectively used to perform PCR with saliva DNA of each of 6 human subjects, and the amplicons of individual PCR reactions were cloned. The phylogenetic analysis showed cloned sequences were all affiliated to *S. parasanguinis*, which further validates the specificity of two primer pairs, and that individual subjects harbored multiple genotypes of *S. parasanguinis* in saliva. By spiking *S. parasanguinis* into human fecal samples, we found the quantification limit of quantitative real-time PCR (qPCR) assays for both primer pairs was 5–6 log_10_ *groEL* copies/g feces. Human fecal *S. parasanguinis* amounts quantified with qPCR using each of the two primer pairs correlated well with those determined with metagenomic sequencing. qPCR with either primer pair showed periodontitis patients had significantly lower level of saliva *S. parasanguinis* than healthy people. In both feces and saliva, the *S. parasanguinis* abundances quantified with two primer pairs exhibited strong and significant correlation. Our results show that the two *S. parasanguinis*-specific primer pairs can be used to quantify and profile human saliva and fecal *S. parasanguinis*.

## Introduction

*Streptococcus parasanguinis* is a common human commensal bacterial species colonizing multiple body sites. It is a prevalent bacteria in the oral cavity of both adults and infants (Franzosa et al., 2014; Dzidic et al., 2018), and plays an important role in dental plaque formation (Garnett et al., 2012) and significantly inhibits the growth the periodontopathogens by producing hydrogen peroxide (Herrero et al., 2016). The abundance of oral *S. parasanguinis* is associated with childhood allergies (Dzidic et al., 2018) and caries (Becker et al., 2002). *S. parasanguinis* is also frequently isolated from the breast milk of women (Lara-Villoslada et al., 2007), and our human breast milk microbiota-associated mouse model showed that breast milk *S. parasanguinis* can colonize gut (Wang et al., 2017). Indeed, *S. parasanguinis* is one of the dominating pioneer colonizers of human infant intestine in first days of life (Songjinda et al., 2005), a predominant bacterial species in the small intestine of adults (van den Bogert et al., 2013b), and also detected in the feces of children (Zhang et al., 2015) and adults (Franzosa et al., 2014). *In vitro* experiments showed one human small intestinal *S. parasanguinis* strain moderately activated NF-κB via TLR2/6 signaling, and thus induced the maturation, activation and cytokine IL12 secretion of human monocyte-derived dendritic cells (van den Bogert et al., 2014). Occasionally, *S. parasanguinis* can translocate to the bloodstream and result in infective endocarditis (Naveen Kumar et al., 2014). Therefore, it is necessary to develop molecular methods to quantify and profile *S. parasanguinis* in human microbiome samples to understand its role in human health and diseases.

16S rRNA gene is often used to identify bacteria at the species level, but the interspecies divergence of *Streptococcus* 16S rRNA gene as low as 0.3% does not allow the effective discrimination of closely related Streptococci (Glazunova et al., 2009). Van den Bogert et al. (2013a) identified unique genes that are solely present in the genome of one *S. parasanguinis* strain isolated from the ileostoma effluent of one healthy Ileostomist, and designed specific PCR primers for the strain, but the primers cannot detect *S. parasanguinis* strains colonizing other human subjects. Park and Kook (2013) developed *S. parasanguinis*-specific PCR primers based on the genomic DNA sequences with unknown function, however, it is unknown whether the unannotated genomic sequences are present in all strains of *S. parasanguinis*.

The housekeeping *groEL* gene is ubiquitously distributed among bacteria. It encodes chaperonin GroEL (synonyms are Cpn60 and Hsp60) that assists proper protein folding in bacterial cells. The *groEL* gene sequences are widely used to study the phylogeny of bacteria (Viale et al., 1994) including *Streptococcus* spp. (Teng et al., 2002; Glazunova et al., 2009; Lourenco et al., 2017; Leigh et al., 2018). In addition, *groEL* gene shows 3.4% divergence among different *Streptococcus* species, and thus has much higher power than 16S rRNA gene in discriminating *Streptococcus* species (Glazunova et al., 2009). The Chaperonin Sequence Database^1^ (Junick and Blaut, 2012) currently contains ∼22,000 *groEL* sequences of prokaryotes, eukaryotes, and archaea, and among these sequences, 866 are from 64 *Streptococcus* species, and 8 are from 7 *S. parasanguinis* strains. This database provides good reference sequences of *groEL* gene for PCR primer development.

In the present study, we developed two PCR primer pairs, which consist of three primers, to specifically detect human commensal *S. parasanguinis*. Both primer pairs were used to amplify and clone the *groEL* gene of human saliva *S. parasanguinis*, and quantify human fecal and saliva *S. parasanguinis* with quantitative real-time PCR (qPCR).

## Materials and Methods

### Design of *groEL* Gene-Targeted *S. parasanguinis*-Specific Primers

The *groEL* sequences of *S. parasanguinis* strains and closely related *Streptococcus* species (Glazunova et al., 2009) were downloaded from the Chaperonin Sequence Database in March 2017^1^ and subjected to a multiple alignment with ClustalX version 2.1 provided by the European Bioinformatics Institute^2^. The region of 552 bp of the complete *groEL* gene named universal target (UT) sequences was used to manually identify the discriminative nucleotides. Three primers, two forward primers Spa146f and Spa93f, and one reverse primer Spa525r were designed, and they composed two primer pairs, Spa146f-Spa525r and Spa93f-Spa525r (Table 1).

**TABLE 1:** Two *S. parasanguinis*-specific primer pairs based on the *groEL* gene*^*a*^.*

**Primer Pair**	**Sequence (5′ to 3′)^*b*^**	**Product size/bp**
Spa146f/Spa525r	AACAATGCGATYCCAGTATCRAG/CTACGACATTAAAGGTACCDCGG	424
Spa93f/Spa525r	TCCGYCGTGGGATTGAGACC/CTACGACATTAAAGGTACCDCGG	474

**^*a*^*The two primer pairs consist of three primers, two forward primers Spa146f and Spa93f, and one reverse primer Spa525r. *^*b*^*IUPAC ambiguity codes: R (A or G), Y (C or T), D (G or A or T).*

The genomes of 30 *S. parasanguinis* strains deposited in the NCBI genome collection database^3^ were downloaded. The sequence of each designed primer was blast against the 30 *S. parasanguinis* genomes using NCBI Basic local alignment search tool^4^. Each primer was also blast against all the *groEL* gene sequences of the Chaperonin Sequence Database^1^ online with the “Primer Blast” program, and then against all sequences in NCBI nucleotide collection (nr/nt) database with the blastn tool^4^.

### Simulated PCR (SPCR)

Eight hundred and sixty six *groEL* universal target (UT) sequences of 64 *Streptococcus* spp., which included 8 sequences of 7 *S. parasanguinis* strains, were downloaded from the Chaperonin Sequence Database^1^ as templates. The sequences of these templates and each of the two primer pairs, Spa146f-Spa525r and Spa93f-Spa525r, were introduced into SPCR (Cao et al., 2005). The product amplification coefficient, increase of which corresponds to the enhancement of the annealing temperature in experimental PCR, was set to 0.80 (the recommended value according to the SPCR developer) and 0.90, respectively, for individual primer pair, and the SPCR algorithm output the template sequences that can be amplified by the tested primer pair under each coefficient.

### Bacterial Strains and Human Subjects

Strains *S. parasanguinis* F278 and *S. salivarius*F286 were previously isolated from human breast milk in our laboratory, and the accession numbers of the 16S rRNA gene sequence of the two strains were KY038191 and KY038192, respectively (Wang et al., 2017). *S. sanguinis* ATCC 10556, *S. mutans* UA159 and *S. gordonii* ATCC 10558 were obtained from the American Type Culture Collection (ATCC). All strains were cultured in liquid M17 medium (Hopebiol, Qingdao, China) in an anaerobic chamber (DG500, Don Whitley Scientific, United Kingdom) at 37°C for 10 h.

The feces of 22 Chinese children, which contained varied abundance of*S. parasanguinis* according to our previous metagenomic sequencing (Zhang et al., 2015) were used in the present study. These 3 to 6-year-old children were diagnosed with Prader–Willi syndrome or simple obesity, and were recruited previously to study the role of gut microbiota in genetic and simple obesity (Zhang et al., 2015). The cohort study was performed under the approval of the Ethics Committee of the School of Life Sciences and Biotechnology, Shanghai Jiao Tong University (No. 2012-016). Written informed consents were obtained from the guardians of the children.

The saliva DNA of 28 newly diagnosed periodontitis patients and 26 periodontally healthy volunteers, which were stored at −80°C after extraction (Chen et al., 2015), were used in the present study. The periodontitis patients aging 29–67 years and the periodontally healthy volunteers aging 21–55 years were all Chinese Han people, and recruited previously to compare the oral microbiota composition by doing illumina sequencing of 16S rRNA gene V3-V4 region (Chen et al., 2015). This study was approved by the Ethics Committee of Shanghai Ninth People’s Hospital affiliated to Shanghai Jiao Tong University, School of Medicine, China (Document No. 201262). Written informed consent was obtained from all the participants.

### DNA Extraction From Bacterial Cultures and Human Feces

Genomic DNA from the bacterial cultures was extracted as described previously (Wang et al., 2017). DNA extraction from fecal samples was conducted as previously described (Godon et al., 1997). Both exaction processes included chemical lysis and bead beating to break the bacterial cells. The integrity of the DNA was assessed by using 0.8% agarose gel electrophoresis gels stained with ethidium bromide, and the concentration was quantified with PicoGreen fluorescent dye (Thermo Fisher Scientific, Sunnyvale, CA, United States) by using SpectraMax M5 microplate reader (Molecular Devices, San Francisco, CA, United States).

### PCR Amplification With Genomic DNA of Bacterial Strains

The two designed primer pairs, Spa146f-Spa525r and Spa93f-Spa525r, were respectively used to do experimental PCR under gradient annealing temperatures using the genomic DNA of *S. parasanguinis* F278, *S. salivarius* F286, *S. sanguinis* ATCC 10556, *S. mutans* UA159 and *S. gordonii* ATCC 10558, respectively, as template. The PCR program included an initial denaturing step of 5 min at 94°C, 30 cycles of 95°C for 30 s, a certain annealing temperature between 58 and 63°C for 20 s, and 72°C for 45 s, and a final extension at 72°C for 7 min. Each 25 μl PCR mixture contained 1 × PCR buffer, 2mM MgCl_2_, 0.2 mM of each dNTP, 0.25 μM of each primer, 0.2 U Taq polymerase (TaKaRa, Dalian, China), and 20 ng of template DNA. Amplifications were performed with the ABI PCR thermal cycler (Applied Biosystems, United States). PCR products were assessed by electrophoresis on 1.5% (wt/vol) agarose gel.

### PCR Amplification and Cloning of *S. parasanguinis groEL* Gene From Human Saliva and Phylogenetic Analysis of Cloned Sequences

PCR was performed with primer pair Spa146f-Spa525r and Spa93f-Spa525r, respectively, using the saliva DNA of each of six human volunteers as template. The 25 μl PCR mixture contained 1 × PCR buffer, 2 mM MgCl_2_, 0.2 mM of each dNTP, 0.25 μM of each primer, 0.2 U Taq polymerase (TaKaRa, Dalian, China), and 2 μl saliva DNA. The PCR program was as follows: 94°C for 5 min; 30 cycles of 95°C for 30 s, 61°C and 62°C for Spa146f-Spa525r and Spa93f-Spa525r, respectively, for 20 s, and 72°C for 45 s; and finally 72°C for 7 min. The size and specificity of PCR products were checked by electrophoresis on 1.5% (wt/vol) agarose gel.

The products of individual PCR reactions were excised from the 1.5% agarose gel and purified using the Gel Extraction Kit 200 (Omega, United States) as recommended by the manufacturer. Purified amplicons were ligated into the pGEM-T easy vector (Promega, Madison, WI, United States), and then transformed into competent *E. coli* DH5α cells. From each library, 15 recombinant clones were randomly selected and sequenced (Life Technologies, Shanghai, China).

The cloned sequences were blast against the nr database of GenBank using the basic local alignment search tool (BLAST)^5^ to determine the closest relative bacteria species. The cloned sequences and the reference *groEL* gene of *Streptococcus* spp. were aligned, and the reference gene sequences were trimmed to the same length of clone sequences. Neighbor-joining phylogenetic trees containing the cloned sequences and the trimmed *groEL* gene of *Streptococcus* spp. were constructed with the Molecular Evolutionary Genetics Analysis package 7 (MEGA 7) using the Maximum Composite Likelihood method. The phylogenetic robustness was assessed by bootstrap analysis with 1000 replicates.

### Quantitative Real-Time PCR of *S. parasanguinis* in Human Feces and Saliva

Quantitative real-time PCR (qPCR) was performed with primer pair Spa93f-Spa525r and Spa146f-Spa525r, respectively, for each human fecal or saliva sample. The PCR was done in 96-well optical plates on LightCycler^®^ 96 Real-Time PCR System sequence detector (Roche, United States). The 20-μl reaction mixture contained 1 × FastStart SYBR green I PCR Mix (iQ^TM^ SYBR^®^ Green, Bio-Rad, United States), 0.5 μM of each primer, and 2 μl fecal/saliva DNA template. The PCR program was as follows: 94°C for 5 min; 40 cycles of 95°C for 30 s, 61°C and 62°C for Spa146f-Spa525r and Spa93f-Spa525r, respectively, for 20 s, 72°C for 45 s, and melting temp 82°C for 5 s for fluorescence detection. To confirm the specificity of the PCR reaction, melting curve analysis was performed after amplification by increasing the temperature at a rate of 0.2°C per second from 65°C to 97°C with continuous fluorescence measurement. PCR reactions were performed in triplicate. Two recombinant plasmids, SH3-7-146f and SH3-9-93f, which were picked from the clone libraries of the *S. parasanguinis groEL* gene of human saliva, were used to construct the standard curve for the qPCR with primer pair Spa146f-Spa525r and Spa93f-Spa525r, respectively. The *S. parasanguinis groEL* gene copy number was quantified using standard curves constructed from known concentrations of the plasmid DNA ranging from 5 × 10^1^ to 5 × 10^8^ copies/μl. The abundance of *S. parasanguinis* was expressed as copies/ng DNA.

### Spiking Experiments

To determine the quantitative limit of the qPCR with the primer pairs, three fecal samples, which was collected from three obese children respectively and contained no *S. parasanguinis* according to metagenomic sequencing (Zhang et al., 2015), were used in the spiking experiments. Aliquots of 200 mg feces were spiked with 10-fold serial dilutions of the culture of *S. parasanguinis* F278 strain ranging from 2 to 9 log_10_ cells. The concentrations of *S. parasanguinis* in the spiked samples were numerated by plate counting on M17 agar medium in triplicate. DNA was extracted from spiked feces as described above.

### Statistics

Data statistics was done with GraphPad Prism version 6.0. Shapiro-Wilk test was used to check the normal distribution of the data. Mann–Whitney U test was used to compare the abundances of saliva *S. parasanguinis* between periodontitis patients and orally healthy people. The Pearson’s correlation test was used to examine the correlation of *S. parasanguinis* abundances in fecal or saliva quantified with different techniques.

### Nucleotide Sequence Accession Numbers

The partial *groEL* gene sequences of *S. parasanguinis* cloned from human saliva were deposited in GenBank under accession numbers MK608386 – MK608559, MK616660 – MK616661 and MK637615 – MK637617.

## Results

### Primer Specificity

Primer Spa146f, Spa93f, and Spa525r showed 100% similarity with the *groEL* gene of all 7 *S. parasanguinis* strains deposited in Chaperonin Sequence Database^6^ (Table 2 and Supplementary Tables S1–S3). Besides, we downloaded the genome sequences of 30 *S. parasanguinis* strains from NCBI genome database^7^, and compared the primer sequences to the *groEL* gene sequence of each of the 30 *S. parasanguinis* strains. Spa146f 100% matched with 28 of 30 *S. parasanguinis* strains, and showed one base mismatch with 2 strains in the middle position closer to 5′ terminal where the mismatches had no significant effect on PCR amplification (Kwok et al., 1990). Spa93f fully matched with 18 of 30 *S. parasanguinis* strains, and had one base mismatch with three strains in the middle position or 5′ end, and had one base mismatch with nine strains at 3′ terminal. Spa525r 100% matched with 27 *S. parasanguinis* strains, and had one base mismatch with three strains in the middle position close to 5′ terminal (Supplementary Tables S1–S3).

**TABLE 2:** The sequence alignment of the *S. parasanguinis*-specific primers with the *groEL* gene of strains of *Streptococcus* spp.

**Species**	**Strains**	**Spa146f (5′ - 3′)**	**Spa93f (5′ - 3′)**	**Spa525r(5′ - 3′)**
		**AACAATGCGATYCCAGTATCRAG**	**TCCGYCGTGGGATTGAGACC**	**CTACGACATTAAAGGTACCDCGG**
*S. parasanguinis*	ATCC 15912	•••••••••••••••••••••••	••••••••••••••••••••	•••••••••••••••••••••••
	ATCC 903	•••••••••••••••••••••••	••••••••••••••••••••	•••••••••••••••••••••••
	FW213	•••••••••••••••••••••••	••••••••••••••••••••	•••••••••••••••••••••••
	M44	•••••••••••••••••••••••	••••••••••••••••••••	•••••••••••••••••••••••
	M688	•••••••••••••••••••••••	••••••••••••••••••••	•••••••••••••••••••••••
	F0405	•••••••••••••••••••••••	••••••••••••••••••••	•••••••••••••••••••••••
	SK236	•••••••••••••••••••••••	••••••••••••••••••••	•••••••••••••••••••••••
*S. australis*	ATCC 700641	•• T ••C••C•••••••• TG • C • A	•••••••••••••••• AG • A	•••••••••• G •• A •• T ••••• A
	M624	•• T ••C••C•••••••• TG • C • A	•••••••••••••••• AG • A	•••••••••• G ••••• T ••••• A
*S. infantis*	ATCC 700779	•••••••• A •••••••• T •• T • A	•••••••••••••••• A •• T	• A •• T •• G •• G •• A •• C ••••• A
	SK1076	•••••••• T ••••• T •• TG • C • A	•••••••••••••••• AG • A	• A •• T •• G •• G •• A •• C ••••• A
*S. oralis*	ATCC 35037	•••••••• C •••••T•• TG • C • A	•••••••••••••••• AG • A	•••••••••• G ••••• T ••••• A
	ATCC 49296	•••••••• C •••••T•• TG • C • A	• T •••••••••••••• AG • T	•••• T •• G •• G ••••• T ••••• A
	CIP 105158	•T• GCC ••A• AG ••• TGT • A •• A	•••••G•• ACCT •• A • TGT •	•••••••••••••••••••••••
	CIP 104985	• T • GCC ••A• AG ••• TGT •A•• A	•••••G•• ACCT •• A • TGT •	•••••••••••••••••••••••
*S. oligofermentans*	AS 1.3089	G •••C•••••••••G•• T •• T • A	•••••••C•••••••• AG • A	• C •• T ••••• G ••••••••••• A
*S. pneumoniae*	ATCC 33400	•••••C••C••••• T •• TG • C • A	• T •••••••••••••• A •• A	•••• T •• G •• G ••••• T ••••• A
	ATCC 27336	•••••C••C••••• T •• TG • C • A	• T •••••••••••••• A •• A	•••• T •• G •• G ••••• T ••••• A
*S. pseudopneumoniae*	SK674	•••••C••C••••• T •• TG • C • A	• T •••••••••••••• AG • A	•••• T •• G •••••••• T ••••• A
	ATCC BAA-960	•••••C••C••••• T •• TG • C • A	• T •••••••••••••• AG • A	•••• T •• G •••••••• T ••••• A
*S. mitis*	ATCC 9811	•••••••• T ••••• T •• TG • C • A	• T •••••••••••••• A •• A	•••• C •• G •• G ••••• T ••••• A
	ATCC 49456	•••••••• C ••••• T•• TG • C • A	• T •••••••••••••• AG • A	• C •• C ••••• G ••••• T ••••• A
*S. acidominimus*	NCTC11291	GCG •T•T• ACA ••• G •• T •• TG •	• T •••••••• T ••••• AG • A	• A •••••••• G •• A •••••••• A
*S. suis*	BM407	GCAC •A••A• G ••• T ••••• C• A	• T ••••• C •••••••• AG ••	• A •• A •• G •• G •• T •••••••• A
	SC84	GCAC •A••A• G ••• T ••••• C• A	• T ••••• C •••••••• AG ••	• A •• A •• G •• G •• T •••••••• A
*S. massiliensis*	M212	• C ••• CAGTG ••••C•• T •• AG •	• T ••••• C •••••••• AG • A	•••••••••• G •• A •• T ••••••
	4401825	• C ••• CAGTG ••••C•• T •• AG •	• T ••••• C •••••••• AG • A	•••••••••• G •• A •• T ••••••
*S. sanguinis*	ATCC 29667	TCT ••CT• AG ••••••• T •• T • A	••••••• C •••••••• AG • A	•••••••••••••••• T ••••• A
	ATCC 10556	TCT ••CT• AG ••••••• T •• T • A	••••••• C •••••••• AG • A	•••••••••••••••• T ••••• A
*S. gordonii*	ATCC 10558	G • A • C •••T••••••••T•• T • A	•••••••••••••••• AG • A	•••• A •• G •• G •• A •• T ••••• A
	M437	G • A • C •••T••••••••T•• T • A	•••••••••••••••• AG • A	•••• A •• G •• G •• A •• C ••••• A
*S. salivarius*	ATCC 7073	GCT •T••• ACA •••••• TG • T • A	•••••••••••••••• AG • A	• A •••••••• G •• A •• T ••••• A
	JIM8777	GC ••T••• TCA •••••• TG • T • A	•••••••••••••••• AG • A	• A •• A ••••• G •• A •• T ••••• A
*S. alactolyticus*	CIP 103244	• T • GTC • TA • AG ••• TGC • T •• A	•••• TG •• ACCT •• A • TGT •	•••••••••••••••••••••••
	ATCC 43077	TCA • T ••• ACAA ••••• TG • T • A	• T ••••• C •• T •• C •• AT • T	•••••••••••••••••••••••

Meanwhile, the sequences of the three primers were compared to the *groEL* gene of *Streptococcus* species other than *S. parasanguinis* (Table 2). The two forward primers, Spa146f and Spa93f, showed multiple mismatches with the sequences of non-*S. parasanguinis* strains within the last five bases at the 3′ terminal of the primer where the mismatches significantly prevent the amplification of non-specific sequences (Kwok et al., 1990). The reverse primer, Spa525r, 100% matched with the *groEL* gene of two (but not all) *S. oralis* strains and two *S. alactolyticus* strains, but had multiple mismatches with other non-*S. parasanguinis* strains.

The three primers were respectively blast against the *groEL* gene sequences of the Chaperonin Sequence Database^6^ using the Primer Blast tool of the database, and this tool calculated the score value by taking into account of identical bases and gaps of two aligned sequences to reflects the identity of the primer sequence with reference sequences (Altschul et al., 1990). The higher the score is, the more identical the primer is with the reference sequence. The two forward primers, Spa146f and Spa93f, had highest score (46 and 40, respectively) with only *S. parasanguinis*, and showed significantly lower scores (no higher than 32 and 34, respectively) with non-*S. parasanguinis* bacteria. The reverse primer, Spa525r, had the highest score 46 with *S. parasanguinis* strains and two *S. alactolyticus* strains, and lower scores (no higher than 44) with other bacterial strains.

Each of the three primers was then subjected to online BLAST analysis against the DNA sequences of the NCBI nucleotide collection (nr/nt) database with the NCBI blastn tool, and the alignment score values were also given to evaluate the identity of the primer sequence with the reference sequences. In accordance with the results of Primer Blast in Chaperonin Sequence Database, Spa146f and Spa93f had highest score (46.1 and 40.1, respectively) with only *S. parasanguinis* but significantly lower scores (no higher than 36.2 for both primers) with non-*S. parasanguinis* bacteria; and Spa525r had the highest score 46.1 with *S. parasanguinis* strains, two *S. alactolyticus* strains, and two *S. oralis* strains, and showed much lower scores (no higher than 38.2) with other non-*S. parasanguinis* strains.

The SPCR algorithm predicts *in silico* whether individual pairs of PCR primers produce amplicons with template DNA sequences at certain product amplification coefficient, increase of which corresponds to the increase of annealing temperature of actual PCR (Cao et al., 2005). The 866 *groEL* gene sequences of 64 *Streptococcus* species, including the 8 sequences of 7 *S. parasanguinis* strains, were downloaded from the Chaperonin Sequence Database, and input as templates into SPCR algorithm. According to the prediction of SPCR, Spa146f-Spa525r amplified only *S. parasanguinis* sequences at the product amplification coefficient 0.8, while Spa93f-Spa525r did so only when the product amplification coefficient was increased to 0.9 (Supplementary Table S4 and Supplementary Figure S1). This suggests that the both primer pairs are capable to specifically amplify the *groEL* gene of *S. parasanguinis* under PCR conditions stringent enough, and that compared to Spa93f-Spa525r, Spa146f-Spa525r requires less stringent condition to achieve specific amplification.

Actual PCR was performed using each of the two primer pairs and the genomic DNA of strains belonging to *S. parasanguinis*, and *S. salivarius*, *S. sanguinis*, *S. mutans*, and *S. gordonii* that are common human commensal streptococci. Spa146f-Spa525r yielded amplicons of the expected size only for *S. parasanguinis* at annealing temperature 58 – 63°C, whereas Spa93f-Spa525r did so at annealing temperatures no less than 62°C (Supplementary Figures S2, S3). Spa93f-Spa525r produced an amplicon with the genomic DNA of *S. salivarius* as templates at annealing temperature 59 – 62°C, but it was about 100 bp smaller in size than *S. parasanguinis* amplicon (Supplementary Figure S3). The lower annealing temperature of Spa146f-Spa525r compared to Spa93f-Spa525r for specific amplification of *S. parasanguinis* under the conventional PCR condition is in accordance with the SPCR prediction. However, Spa93f-Spa525r did not generate any amplicon with the *S. salivarius* genomic DNA as templates in qPCR assays in which the annealing temperature was 62°C (Supplementary Figure S4).

### PCR-Cloning of the *S. parasanguinis* groEL Gene in Human Saliva Samples With the Primer Pairs Spa146f-Spa525r and Spa93f-Spa525r

In order to further evaluate the specificity of the two primer pairs, we constructed clone libraries with PCR products amplified from the saliva DNA with primer pair Spa146f-Spa525r and Spa93f-Spa525r, respectively, for each of 6 people (Supplementary Figure S5). Saliva samples were used because they were reported to harbor diverse and abundant *Streptococcus* spp. (Sakamoto et al., 2000; Belstrom et al., 2017).

Fifteen clones were randomly selected from each library and sequenced. The Blastn analysis against the DNA sequences of the nucleotide collection (nr/nt) database of NCBI showed that the nearest neighbor bacteria of all the sequenced clones obtained with Spa146f-Spa525r and Spa93f-Spa525r were *S. parasanguinis*, with the similarity being 95–100%.

**FIGURE 1:**
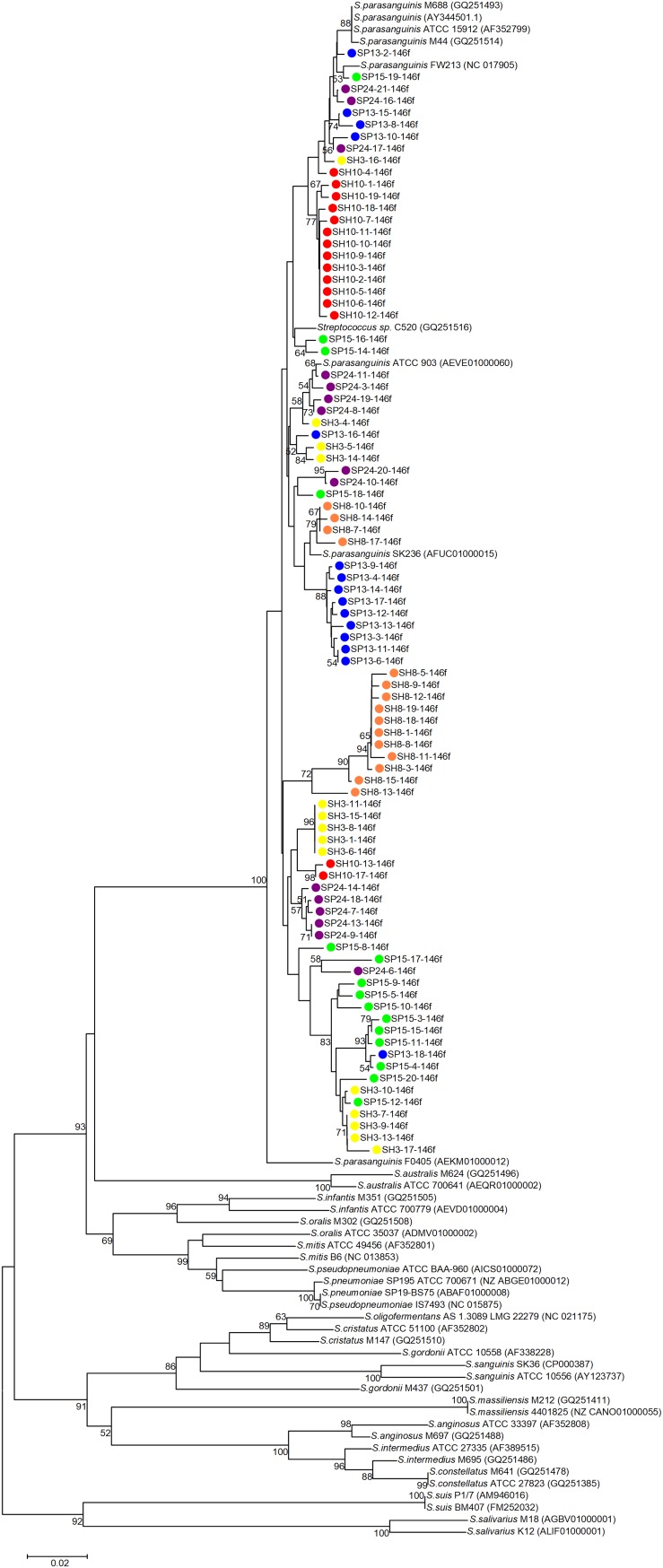
Phylogenetic tree of the *groEL* gene fragments cloned by the primer pair Spa146f-Spa525r from the saliva of six human subjects and those of known *Streptococcus* species. The cloned sequences of the present study are labeled by dots of varied colors, and clones of the identical color are from the saliva sample of one person. Strains of known *Streptococcus* species retrieved from the GenBank are indicated by italics, and the accession numbers of their *groEL* genes are given in parentheses following the bacterial names. Bootstrap values greater than 50% are indicated at the nodes.

**FIGURE 2:**
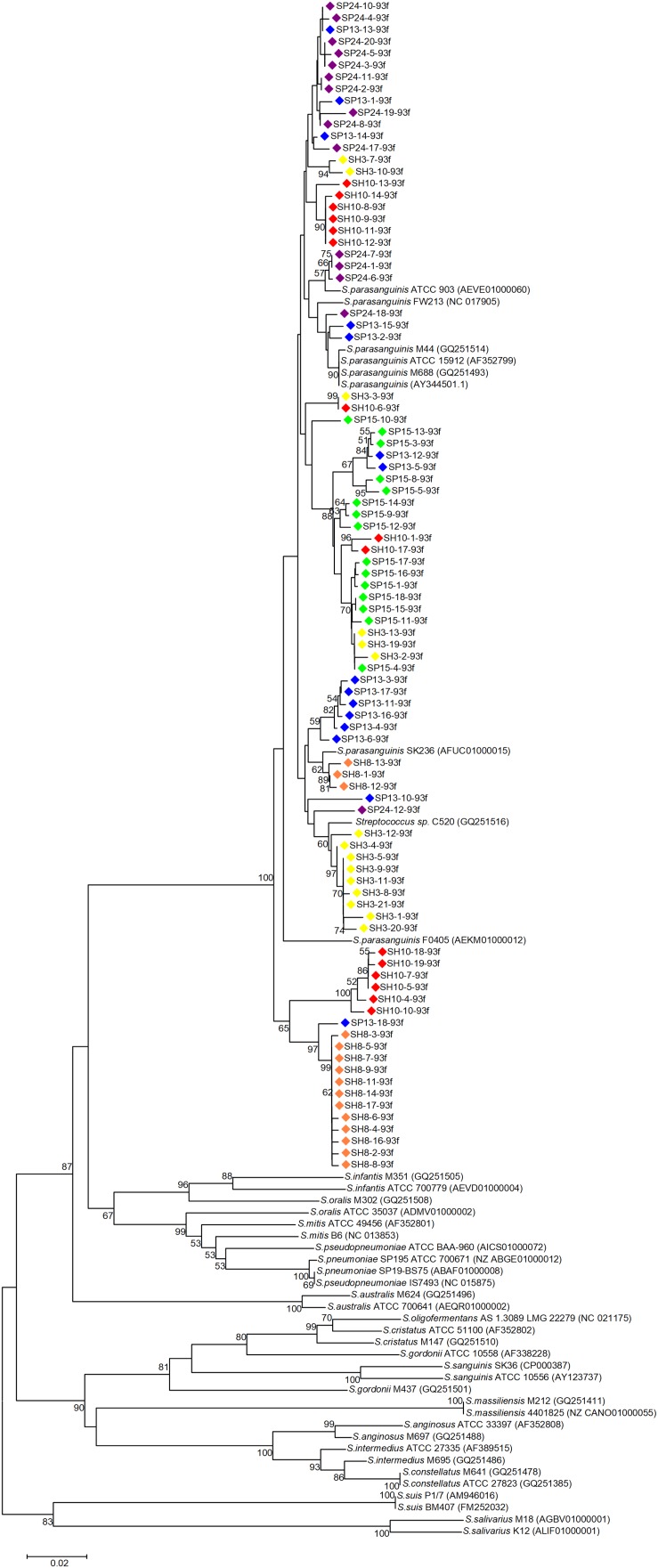
Phylogenetic tree of the *groEL* gene fragments cloned by the primer pair Spa93f-Spa525r from the saliva of six human subjects and those of known *Streptococcus* species. The cloned sequences of the present study are labeled by dots of varied colors, and clones of the identical color are from the saliva sample of one person. Strains of known *Streptococcus* species retrieved from the GenBank are indicated by italics, and the accession numbers of their *groEL* genes are given in parentheses following the bacterial names. Bootstrap values greater than 50% are indicated at the nodes.

Phylogenetic trees were constructed with the cloned sequences obtained with primer pair Spa146f-Spa525r and Spa93f-Spa525r, respectively (Figures 1, 2). Regardless of the primer pair, all cloned sequences clustered with known *S. parasanguinis* strains, and separated from other *Streptococcus* spp. In both trees, the *S. parasanguinis* sequences, including cloned sequences and reference sequences of known *S. parasanguinis* isolates, formed different lineages, and the cloned sequences from the same human subjects distributed in at least two different lineages (Figures 1, 2, and Supplementary Figure S6).

**FIGURE 3:**
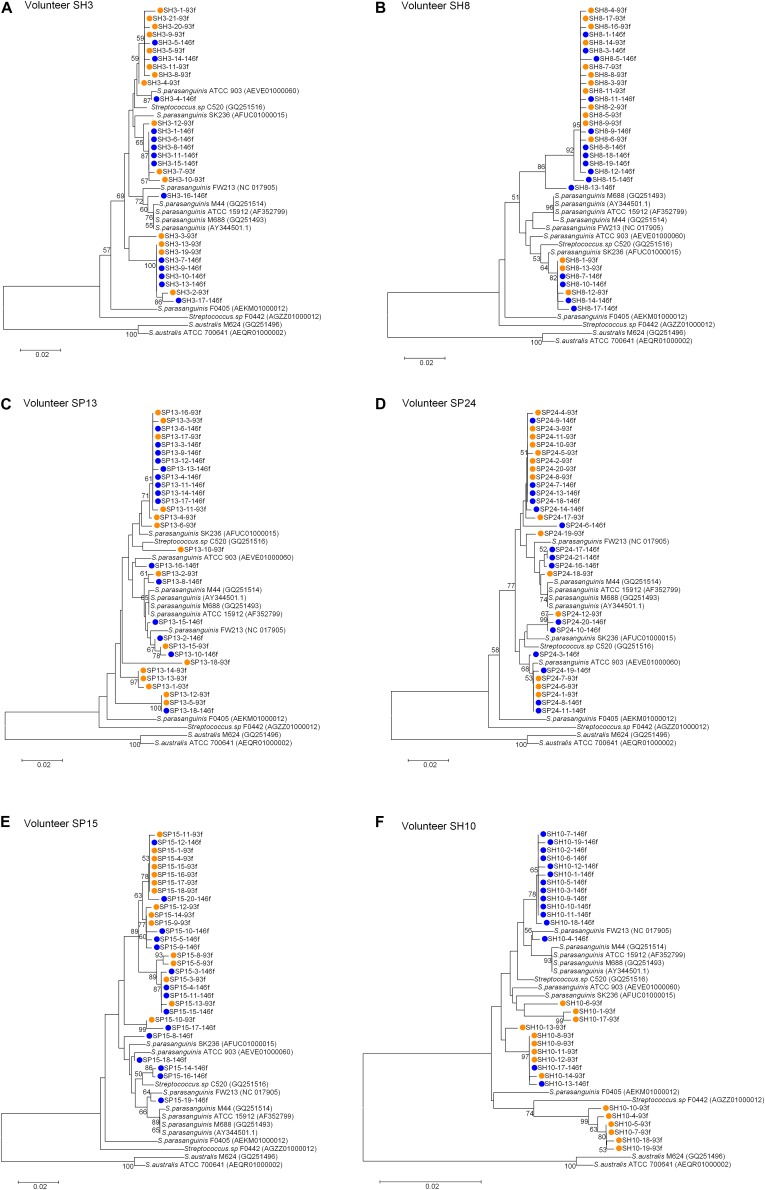
Phylogenetic trees of the *groEL* gene fragments cloned by the primer pair Spa146f-Spa525r and Spa93f-Spa525r from the saliva of individual human subjects and those of known *Streptococcus* species. The sequences cloned with Spa146f-Spa525r are labeled with blue dots, and those cloned with Spa93f-Spa525r are labeled with orange dots. Bootstrap values greater than 50% are indicated at the nodes. **(A)** The tree of sequences cloned from the saliva of human subject SH3; **(B)** The tree of sequences cloned from the saliva of human subject SH8; **(C)** The tree of sequences cloned from the saliva of human subject SP13; **(D)** The tree of sequences cloned from the saliva of human subject SP24; **(E)** The tree of sequences cloned from the saliva of human subject SP15; **(F)** The tree of sequences cloned from the saliva of human subject SH10.

For each of the six people, the sequences cloned with both primer pairs were aligned, and the Spa93f-Spa525r sequences were trimmed so that they are of the same length with Spa146f-Spa525r sequences, and then the processed sequences of both primer pairs were used to build the phylogenetic tree (Figure 3). For four people, the sequences cloned with the two primer pairs distributed among one another in the tree (Figures 3A–D). For one person (SP15), 10 of 15 Spa146f-Spa525r sequences and all Spa93f-Spa525r sequences distributed among one another, and five Spa146f-Spa525r sequences located in clusters distinct from others (Figure 3E). For only one person (SH10), the Spa146f-Spa525r sequences and Spa93f-Spa525r sequences formed separate lineages (Figure 3F). This indicates that in a few but not all humans, primer pair Spa146f-Spa525r and Spa93f-Spa525r may preferentially detect different *S. parasanguinis* strains.

### The Standard Curve and Quantification Limit of qPCR Assay of *S. parasanguinis*

Ten-fold serial dilutions of pGEM-T Easy plasmids containing partial *groEL* gene sequence of *S. parasanguinis* were used to generate the standard curve for primer pair Spa146f-Spa525r and Spa93f-Spa525r. In the range of 1 × 10^2^ to 1 × 10^9^ copies per PCR, the standard curves for both primer pairs were highly linear with the *R*^2^ > 0.99. The PCR efficiency was 79–96% for the two primer pairs.

To determine the lowest *groEL* gene copy number that can be detected with the two primer pairs in qPCR assay, fecal samples of three persons, which contained no *S. parasanguinis* as shown by fecal metagenomic sequencing (Zhang et al., 2015), were spiked with 10-fold serial dilutions of *S. parasanguinis* cells. The DNA extracted from the spiked feces was used as templates in qPCR with each of the two primer pairs. For both primer pairs, the quantification limit in feces was 5–6 log_10_ *groEL* copies/g feces depending on the PCR efficiency. When the PCR efficiency was higher than 90%, the limit was 5 log_10_ *groEL* copies/g feces. When the PCR efficiency was between 80 and 90%, the limit became 6 log_10_ *groEL* copies/g feces.

### Quantification of *S. parasanguinis* in Human Fecal Samples by Using qPCR With the Primer Pairs Spa146f-Spa525r and Spa93f-Spa525r

Twenty-two fecal samples that contained varied amounts of *S. parasanguinis* according to our previous metagenomic sequencing (Zhang et al., 2015) were selected, and *S. parasanguinis* in these samples were re-quantified as *groEL* gene copies/ng DNA with qPCR using primer pair Spa146f-Spa525r and Spa93f-Spa525r, respectively (Supplementary Table S5). The melting curves of fecal qPCR products of both primer pairs showed there was no non-specific amplicon and only amplicons corresponding to *S. parasanguinis* were quantified at the fluorescence reading temperature 82°C (Supplementary Figures S7A,B). Because Spa93f-Spa525r produced an amplicon with the genomic DNA of *S. salivarius* as templates under convention PCR condition (Supplementary Figure S3), the fecal qPCR products of Spa93f-Spa525r were also run on 1.5% agarose gel to check the specificity, and there was no non-specific amplicon (Supplementary Figure S7C).

**FIGURE 4:**
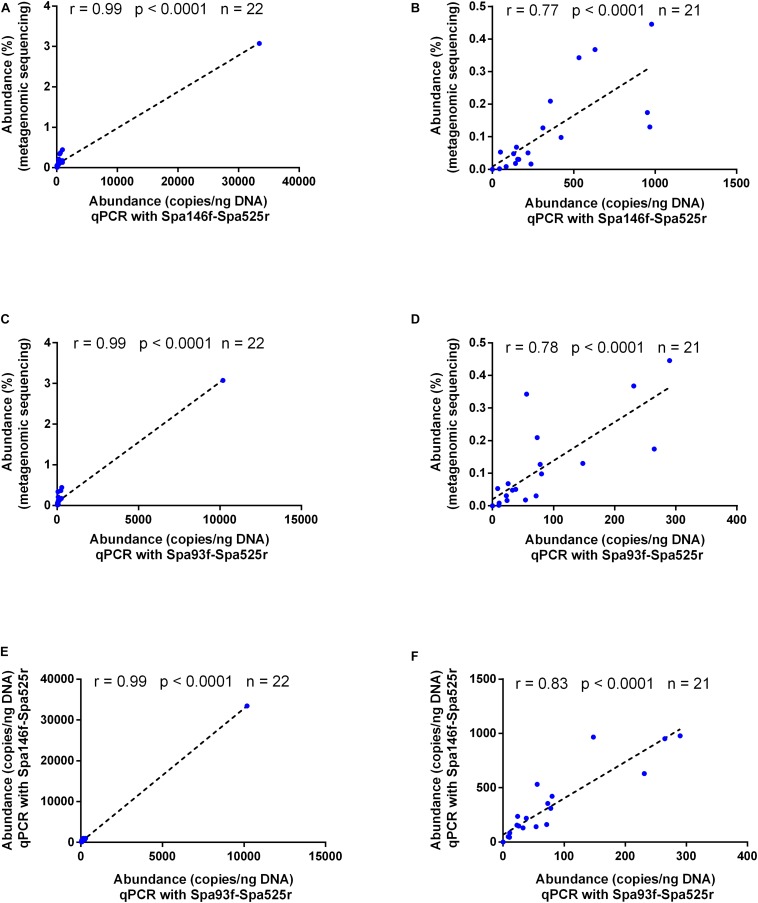
Bivariate scatterplots showing the Pearson’s correlation of the human fecal *S. parasanguinis* concentrations determined by metagenomic sequencing, qPCR with primer pair Spa146f-Spa525r, and qPCR with primer pair Spa93f-Spa525r. The correlation coefficient (*r*), *p*-value, and the number of fecal samples (*n*) are shown in each plot. The relative abundances of *S. parasanguinis* in the metagenomic sequencing dataset were calculated using MetaPhlAn (Segata et al., 2012) in our previous study (Zhang et al., 2015). **(A,C,E)** The correlation among the data of all 22 tested fecal samples. **(B,D,F)** The correlation among the data after the removal of one fecal sample containing extraordinarily higher level of *S. parasanguinis* than other samples.

As shown by Pearson’s correlation analyses, the fecal *S. parasanguinis* abundances measured with either primer pair were well correlated with those obtained with metagenomic sequencing (*r* = 0.99 and *p* < 0.0001 for both Spa146f-Spa525r and Spa93f-Spa525r, Figures 4A,C), and data obtained with the two primer pairs also showed strong and significant correlation (*r* = 0.99 and *p* < 0.0001, Figure 4E). After the removal of the sample that had extraordinarily higher level of *S. parasanguinis* than any other samples, the correlations were still strong and significant among the results of metagenomic sequencing, qPCR with Spa146f-Spa525r, and qPCR with Spa93f-Spa525r (r was between 0.77 and 0.83, and *p* < 0.0001, Figures 4B,D,F).

### Quantification of *S. parasanguinis* in Human Saliva Samples by Using qPCR With the Primer Pairs Spa146f-Spa525r and Spa93f-Spa525r

**FIGURE 5:**
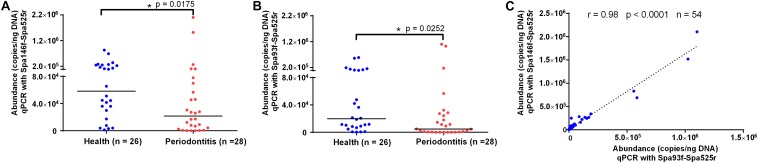
The saliva *S. parasanguinis* concentrations determined by qPCR with two primer pairs in periodontitis patients and periodontally healthy people. **(A)** The saliva *S. parasanguinis* abundance quantified with Spa146f-Spa525r in oral health and periodontitis. **(B)** The saliva *S. parasanguinis* abundance quantified with Spa93f-Spa525r in oral health and periodontitis. The line among the dots represents the median. The values were compared between two groups using the Mann–Whitney *U* test. **(C)** Pearson’s correlation of the human saliva *S. parasanguinis* concentrations determined by qPCR with two primer pairs, Spa146f-Spa525r and Spa93f-Spa525r. The correlation coefficient (*r*), *p*-value, and the number of fecal samples (*n*) are shown.

qPCR using Spa146f-Spa525r and Spa93f-Spa525r, respectively, was performed to quantify the abundances of saliva *S. parasanguinis* in 26 healthy people and 28 periodontitis patients (Supplementary Table S6). The melting curves of saliva qPCR products of both primer pairs showed there was no non-specific amplicon and only amplicons corresponding to *S. parasanguinis* were quantified at the fluorescence reading temperature 82°C (Supplementary Figures S8A,B). The 1.5% agarose gel of saliva qPCR products of Spa93f-Spa525r showed no non-specific amplicon was generated (Supplementary Figure S8C). No matter which primer pairs were used, the results showed that healthy people had significantly higher level (2.7 fold for Spa146f-Spa525r and 4.2 fold for Spa93f-Spa525r) of saliva *S. parasanguinis* than periodontitis patients (Figures 5A,B).

The saliva *S. parasanguinis* quantity obtained with the two primer pairs showed strong and significant correlation (*r* = 0.98, *p* < 0.0001, Figure 5C).

## Discussion

In the present study, two pairs of PCR primers that specifically target the *groEL* gene of *S. parasanguinis*, Spa146f-Spa525r and Spa93f-Spa525r, were designed and validated. In many previous studies that developed PCR primers specific for certain bacterial taxa, PCR assays were performed with the genomic DNA of dozens of bacterial strains belonging to targeted and non-targeted taxa as the templates, and the specificity of the primer pairs were validated by the results that only targeted bacteria produced amplicons of expected size (Junick and Blaut, 2012; Leigh et al., 2018). However, this requires rich collection of cultures of bacterial strains of relevant taxa, which not every lab is capable to have. We used an alternative strategy, which made use of large amounts of reference DNA sequences of large-scaled public databases and combined *in silico* PCR simulation with experimental PCR-clone library and sequencing, to evaluate the specificity of the primer pairs. We first blast the primer sequences against DNA sequences deposited in public databases, including the genomes of 30 *S. parasanguinis* strains from the NCBI genome database^8^, the ∼23,000 *groEL* gene sequences of prokaryotes, eukaryotes, and archaea of the Chaperonin Sequence Database^9^ (Junick and Blaut, 2012), and 49,976,402 non-redundant DNA sequences of the NCBI nucleotide collection (nr/nt). Then, we perform *in silico* PCR simulation (Cao et al., 2005) with individual primer pairs and the 866 *groEL* gene sequences of 64 *Streptococcus* species downloaded from the Chaperonin Sequence Database as templates, and confirmed the simulating results by doing experimental PCRs in which the genomic DNA of a few human commensal *Streptococcus* strains was used as the templates. Finally, Spa146f-Spa525r and Spa93f-Spa525r were respectively used to amplify and clone *S. parasanguinis groEL* gene from 6 human saliva samples that contain complex streptococci populations, and phylogenetic analyses were performed to check whether the cloned amplicons were affiliated to *S. parasanguinis*. Our results suggest that the two primer pairs can specifically detect *S. parasanguinis*.

The quantitative results of qPCR assays that were respectively generated with Spa146f-Spa525r and Spa93f-Spa525r were highly consistent. In both human feces and saliva, the *S. parasanguinis* quantity determined with the two primer pairs showed strong and significant correlation. The levels of human fecal *S. parasanguinis* quantified with each of the two primer pairs correlated well with those determined with metagenomic sequencing of fecal total DNA that is independent of PCR amplification. In addition, qPCR using either primer pair showed periodontitis patients had significantly lower level of saliva *S. parasanguinis* than healthy people. These results suggest that both Spa146f-Spa525r and Spa93f-Spa525r can be used to quantify the human fecal and saliva *S. parasanguinis*. Compared to Spa93f-Spa525r, Spa146f-Spa525r required less stringent condition as predicted by *in silico* PCR simulation and lower annealing temperature as shown by experimental PCR to achieve specific amplification, therefore, we suggest to use Spa146f-Spa525r in the case that there are non-specific amplicons and PCR specificity needs to be improved by enhancing annealing temperature. The qPCR using the two primer pairs can be used to detect and quantify *S. parasanguinis* in clinical specimen without bacterial culturing, and this can accelerate the pathogen identification for *S. parasanguinis*-caused diseases or the determination of the association of *S. parasanguinis* with diseases.

In this study, the *S. parasanguinis groEL* gene sequences cloned from human saliva formed different clusters in the phylogenetic tree, and those of known *S. parasanguinis* isolates scattered in these clusters, suggesting the genotype diversity at sub-species level of *S. parasanguinis*. For individual human subjects, the cloned *S. parasanguinis* sequences distributed in varied clusters, indicating that the one person harbored multiple genotypes of *S. parasanguinis* in oral cavity. This is in accordance with previous observations that individual humans harbored more than one genotypes of *S. mutans* (Cheon et al., 2011; Zhou et al., 2011) and S. *oralis* (Do et al., 2009) in oral cavity and multiple genotypes of *S. salivarius* in small intestine (van den Bogert et al., 2013b). Considering that Spa146f-Spa525r and Spa93f-Spa525r may preferentially detect different *S. parasanguinis* strains in some people, we suggest that both primer pairs be used when researchers aim to profile the strain-level diversity of *S. parasanguinis* in complicated bacterial communities. Using the two primer pairs, the *S. parasanguinis groEL* gene could be cloned and profiled from samples of patients (e.g., periodontitis, and infective endocarditis) and from multiple body locations (e.g., breast milk, intestine, skin, and oral cavity) of healthy peoples. The phylogenetic analyses on the cloned sequences could be performed to see whether certain clade(s) of *S. parasanguinis* strains are associated with the diseases or body locations, which help explore the evolution of *S. parasanguinis* in different ecological environments.

The design of bacterial species-specific PCR primers depends on both the strong power of target genes to discriminate phylogenetically close species and as many as possible reference sequences that are of good quality and cover species from a broad range of phylogeny. A good reference sequence database, which collects, curates, and annotates the relevant sequences from diverse species and records the source organisms, can greatly facilitate the primer design. Researchers investigated several conserved house-keeping genes alternative to 16S rRNA gene to differentiate *Streptococcus* species, including *groEL* (Glazunova et al., 2009), *rpoB* (Glazunova et al., 2009), *gyrB* (Itoh et al., 2006; Glazunova et al., 2009), *rpoA* (Hee Kuk et al., 2010), *soda* (Glazunova et al., 2009), *dnaJ* (Itoh et al., 2006), *zwf* and *gki* (Pattarachai et al., 2005), and these genes, except *sodA*, showed better discriminating power than the 16S rRNA gene. Glazunova et al. (2009) found that the minimal sequence divergence among different *Streptococcus* spp. was 0.3, 2.7, 0, 2.5, and 3.4% for the 16S rRNA gene, *rpoB*, *sodA*, *gyrB*, and *groEL*, respectively, and concluded that *groEL* gene represented the best tool for the identification phylogenetic analysis of *Streptococcus* species and subspecies. When we started to design the *S. parasanguinis*-specific primers in 2016, The cpnDB Database^10^ (Hill et al., 2004) had already contained ∼22,000 *groEL* sequences of prokaryotes, eukaryotes, and archaea, and among these sequences, 866 were from 64 *Streptococcus* species, and 8 were from 7 *S. parasanguinis* strains. Besides, the cpnDB sequences are manually curated to ensure high quality entries. cpnDB is still being updated, and currently contains over 25,000 groEL sequence records of prokaryotes, eukaryotes, and archaea, and includes over 4,000 records from bacterial type strains sequences (Vancuren and Hill, 2019). The ICB database for bacterial *gyrB* gene was published in 2001 (Watanabe et al., 2001), but cannot be publicly accessed since 2012. There was no publicly available database for other bacterial house-keeping genes mentioned above in 2016. Considering the high divergency of *groEL* gene sequences among *Streptococcus* species, and the cpnDB Database that provides rich *groEL* reference sequences of high quality, we designed the two primer pairs targeting only *groEL* gene but not two different genes. *groEL* gene has been used to design PCR primers specific for other bacterial species (Junick and Blaut, 2012; Hossain et al., 2013; Ahmed et al., 2015; Hung et al., 2019). Very recently in July 2019, Ogier et al. (2019) published a reference *rpoB* database including 45,000 *rpoB* sequences retrieved from 47,175 genomes sequences from the Integrated Microbial Genomes (IMG) database. Therefore, in the future, it is necessary to design *S. parasanguinis*-specific PCR primer pair(s) targeting *rpoB* gene, and use them together with our *groEL* gene-targeting primer pairs to identify and quantify *S. parasanguinis* in human microbiome samples with minimal chances of obtaining false positive and/or false negative results.

We did *S. parasanguinis*-specific qPCR assays for the saliva samples collected from 28 periodontitis patients and 26 periodontally healthy people of Chinese Han ethnic group, and observed that periodontitis patients had significantly lower level of *S. parasanguinis* than healthy people in saliva. Belstrom et al. (2016, 2017) performed Illumina sequencing of barcoded 16S rRNA gene, and metagenomic and metatranscriptomic sequencing, respectively, for the saliva samples of 10 periodontitis patients and 10 orally healthy individuals from Denmark. In the 16S rRNA gene sequencing dataset, although *S. parasanguinis* could not be quantified at the species level due to the high similarity among the 16S rRNA genes of different *Streptococcus* species, Belstrom et al. (2016) found significantly lower relative abundance of the whole *Streptococcus* genus in periodontitis patients compared to healthy controls; in the Metagenomic and metatranscriptomic datasets, the authors did not detect the difference in saliva *S. parasanguinis* proportion between periodontitis and oral health (Belstrom et al., 2017). In contrast, when the saliva microbiota composition was compared between 139 chronic periodontitis patients and 447 healthy people of the Danish Scandinavian population using Human Oral Microbe Identification Microarray, researchers reported significantly higher level of saliva *S. parasanguinis* in periodontitis subjects (Belstrom et al., 2014). These inconsistent observations of the effect of periodontitis on saliva *S. parasanguinis* abundance may be explained by the varied human subjects recruited, cohort sizes, and techniques used to quantify the bacteria in different studies. In the subgingival plaque microbiota, some studies reported that *S. parasanguinis* was less prevalent in healthy people than in refractory periodontitis patients, and thus probably play an etiological role in periodontitis(Colombo et al., 2009; Colombo et al., 2012; Fine et al., 2013; Duan et al., 2016).

In the future, to better understand the role of oral *S. parasanguinis* in periodontitis, both multi-centered case-control and longitudinal cohort studies should be performed, and *S. parasanguinis* amounts at different oral locations (the saliva, subgingival plaque, and supragingival plaque) can be quantified with qPCR using our PCR primer pairs, and subsequently correlated with the disease and disease severity. The two types of cohort studies must be well controlled by recruiting periodontitis patients of different ages, genders, geographic locations, and ethnic groups, together with the orally healthy individuals matched with the above factors, and people with diseases other than periodontitis should be excluded. Besides, periodontitis severity must be characterized with multiple clinical parameters, including Gingival margin position (GMP), probing pocket depth (PD), attachment loss (AL), and bleeding on probing (BOP), etc. In the case-control cohorts, the amounts of oral *S. parasanguinis* can be compared between periodontitis and oral health, and among periodontitis of different severity. In the longitudinal studies, the *S. parasanguinis* quantity can be monitored at different time points as the periodontitis improves or relapses after medical treatments, and as the originally orally healthy people develop periodontitis. By doing so, the association of oral *S. parasanguinis* at specific oral locations with periodontitis will be demonstrated with least confounding factors, and whether periodontitis changes the distribution of *S. parasanguinis* at different oral locations will be clarified. Furthermore, *S. parasanguinis* strains should be isolated from oral samples of periodontitis patients, and orally inoculated into animal models for periodontitis (David et al., 2010; Oz and Puleo, 2011; Jung et al., 2019) to test their capability to directly influence (alleviate or predispose) periodontitis or affect (inhibit or promote) the pathogenesis of periodontal pathogens, and the quantity of *S. parasanguinis* in the animals can be monitored with the our qPCR method to confirm their survival or study their ecological interactions with the pathogens.

## Conclusion

We developed two pairs of *S. parasanguinis*-specific PCR primers based on the housekeeping *groEL* gene sequences, and used the primers to detect and quantify human oral and fecal *S. parasanguinis* by qPCR. The method described here can be used to monitor the response of *S. parasanguinis* to dietary intervention, medication, and diseases, etc., and provide insights on the role of human commensal *S. parasanguinis* in health and disease.

## Data Availability Statement

Publicly available datasets were analyzed in this study. This data can be found here: http://www.cpndb.ca/ and https://www.ncbi.nlm.nih.gov/.

## Ethics Statement

This study was carried out in accordance with the recommendations of the Ethics Committee of the School of Life Sciences and Biotechnology, Shanghai Jiao Tong University and the Ethics Committee of Shanghai Ninth People’s Hospital affiliated to Shanghai Jiao Tong University, School of Medicine, China with written informed consent from all subjects. All subjects gave written informed consent in accordance with the Declaration of Helsinki. The protocol was approved by the Ethics Committee of the School of Life Sciences and Biotechnology, Shanghai Jiao Tong University (No. 2012-016) and the Ethics Committee of Shanghai Ninth People’s Hospital affiliated to Shanghai Jiao Tong University, School of Medicine, China (Document No. 201262).

## Author Contributions

JS and QC designed the study. QC and SL performed the experiments. GW, HC, and LZ provided sample materials. HL coordinated the bioinformatics analysis. CZ, XP, and LW coordinated the laboratory management. JS and QC wrote and revised the manuscript.

## Conflict of Interest

The authors declare that the research was conducted in the absence of any commercial or financial relationships that could be construed as a potential conflict of interest.

## Footnotes

1 http://www.cpndb.ca

2 http://www.ebi.ac.uk

3 https://www.ncbi.nlm.nih.gov/

4 http://www.ncbi.nlm.nih.gov/BLAST/

5 http://www.ncbi.nlm.nih.gov/Blast

6 http://www.cpndb.ca

7 https://www.ncbi.nlm.nih.gov/genome

8 https://www.ncbi.nlm.nih.gov/genome/microbes/

9 http://www.cpndb.ca

10 http://www.cpndb.ca

## References

[B1] Ahmed R. Rafiquzaman S. M. Hossain M. T. Lee J. M. Kong I. S. (2015). Species-specific detection of Vibrio alginolyticus in shellfish and shrimp by real-time PCR using the groEL gene. *Aqua. Int.* 24 157–170. 10.1007/s10499-015-9916-5

[B2] Altschul F. S. Warren G. Miller W. Myers E. W. Lipman D. J. (1990). Basic local alignment search tool. *J. Mol. Biol.* 215 403–410. 10.1006/jmbi.1990.9999 2231712

[B3] Becker M. R. Paster B. J. Leys E. J. Moeschberger M. L. Kenyon S. G. Galvin J. L. (2002). Molecular analysis of bacterial species associated with childhood caries. *J. Clin. Microbiol.* 40 1001–1009. 10.1128/jcm.40.3.1001-1009.2002 11880430 PMC120252

[B4] Belstrom D. Constancias F. Liu Y. Yang L. Drautz-Moses D. I. Schuster S. C. (2017). Metagenomic and metatranscriptomic analysis of saliva reveals disease-associated microbiota in patients with periodontitis and dental caries. *NPJ Biofilms Microb.* 3:23. 10.1038/s41522-017-0031-34 28979798 PMC5624903

[B5] Belstrom D. Fiehn N. E. Nielsen C. H. Kirkby N. Twetman S. Klepac-Ceraj V. (2014). Differences in bacterial saliva profile between periodontitis patients and a control cohort. *J. Clin. Periodontol.* 41 104–112. 10.1111/jcpe.12190 24303924

[B6] Belstrom D. Paster B. J. Fiehn N. E. Bardow A. Holmstrup P. (2016). Salivary bacterial fingerprints of established oral disease revealed by the human oral microbe Identification using next generation sequencing (HOMINGS) technique. *J. Oral. Microbiol.* 8:30170. 10.3402/jom.v8.30170 26782357 PMC4717152

[B7] Cao Y. Wang L. Xu K. Kou C. Zhang Y. Wei G. (2005). Information theory-based algorithm for in *silico* prediction of PCR products with whole genomic sequences as templates. *BMC Bioinformatics* 6:190. 10.1186/1471-2105-6-190 16042814 PMC1183192

[B8] Chen H. Liu Y. Zhang M. Wang G. Qi Z. Bridgewater L. (2015). A *Filifactor alocis*-centered co-occurrence group associates with periodontitis across different oral habitats. *Sci. Rep.* 5:9053. 10.1038/srep09053 25761675 PMC4356962

[B9] Cheon K. Moser S. A. Whiddon J. Osgood R. C. Momeni S. Ruby J. D. (2011). Genetic diversity of plaque mutans streptococci with rep-PCR. *J. Dent. Res.* 90 331–335. 10.1177/0022034510386375 21297016 PMC3088998

[B10] Colombo A. P. Bennet S. Cotton S. L. Goodson J. M. Kent R. Haffajee A. D. (2012). Impact of periodontal therapy on the subgingival microbiota of severe periodontitis: comparison between good responders and individuals with refractory periodontitis using the human oral microbe identification microarray. *J. Periodontol.* 83 1279–1287. 10.1902/jop.2012.110566 22324467 PMC3971922

[B11] Colombo A. P. Boches S. K. Cotton S. L. Goodson J. M. Kent R. Haffajee A. D. (2009). Comparisons of subgingival microbial profiles of refractory periodontitis, severe periodontitis, and periodontal health using the human oral microbe identification microarray. *J. Periodontol.* 80 1421–1432. 10.1902/jop.2009.090185 19722792 PMC3627366

[B12] David P. Asaf W. Lior S. Amal H. Dita G. Weiss E. I. (2010). Mouse model of experimental periodontitis induced by *Porphyromonas gingivalis*/*Fusobacterium nucleatum* infection: bone loss and host response. *J. Clin. Periodontol.* 36 406–410. 10.1111/j.1600-051x.2009.01393.x 19419440

[B13] Do T. Jolley K. A. Maiden M. C. Gilbert S. C. Clark D. Wade W. G. (2009). Population structure of *Streptococcus oralis*. *Microbiology* 155(Pt 8), 2593–2602. 10.1099/mic.0.027284-27280 19423627 PMC2885674

[B14] Duan D. Scoffield J. A. Zhou X. Wu H. (2016). Fine-tuned production of hydrogen peroxide promotes biofilm formation of *Streptococcus parasanguinis* by a pathogenic cohabitant *Aggregatibacter actinomycetemcomitans*. *Environ. Microbiol.* 18 4023–4036. 10.1111/1462-2920.13425 27348605 PMC5118171

[B15] Dzidic M. Abrahamsson T. Artacho A. Collado M. C. Mira A. Jenmalm M. C. (2018). Oral microbiota maturation during the first 7 years of life in relation to allergy development. *Allergy* 73 2000–2011. 10.1111/all.13449 29602225

[B16] Fine D. H. Kenneth M. Karen F. Debbie T. B. Javier F. David F. (2013). A consortium of *Aggregatibacter actinomycetemcomitans*, *Streptococcus parasanguinis*, and *Filifactor alocis* is present in sites prior to bone loss in a longitudinal study of localized aggressive periodontitis. *J. Clin. Microbiol.* 51 2850–2861. 10.1128/JCM.00729-13 23784124 PMC3754677

[B17] Franzosa E. A. Morgan X. C. Segata N. Waldron L. Reyes J. Earl A. M. (2014). Relating the metatranscriptome and metagenome of the human gut. *Proc. Natl. Acad. Sci. U.S.A.* 111 E2329–E2338. 10.1073/pnas.1319284111 24843156 PMC4050606

[B18] Garnett J. A. Simpson P. J. Taylor J. Benjamin S. V. Tagliaferri C. Cota E. (2012). Structural insight into the role of *Streptococcus parasanguinis* Fap1 within oral biofilm formation. *Biochem. Biophys. Res. Commun.* 417 421–426. 10.1016/j.bbrc.2011.11.131 22166217 PMC3518267

[B19] Glazunova O. O. Raoult D. Roux V. (2009). Partial sequence comparison of the *rpoB, sodA, groEL* and *gyrB* genes within the genus *Streptococcus*. *Int. J. Syst. Evol. Microbiol.* 59(Pt 9), 2317–2322. 10.1099/ijs.0.005488-5480 19620365

[B20] Godon J.-J. Zumstein E. Dabert P. Habouzit F. Moletta R. (1997). Molecular microbial diversity of an anaerobic digestor as determined by small-subunit rDNA sequence analysis. *Appl. Environ. Microbiol.* 63 2802–2813. 9212428 10.1128/aem.63.7.2802-2813.1997 PMC168577

[B21] Hee Kuk P. Jang Won Y. Jong Wook S. Jae Yeol K. Wonyong K. (2010). rpoA is a useful gene for identification and classification of *Streptococcus pneumoniae* from the closely related viridans group streptococci. *Fems Microbiol. Lett.* 305 58–64. 10.1111/j.1574-6968.2010.01913.x 20158524

[B22] Herrero E. R. Slomka V. Bernaerts K. Boon N. Hernandez-Sanabria E. Passoni B. B. (2016). Antimicrobial effects of commensal oral species are regulated by environmental factors. *J. Dent.* 47 23–33. 10.1016/j.jdent.2016.02.007 26875613

[B23] Hill J. E. Penny S. L. Crowell K. G. Goh S. H. Hemmingsen S. M. (2004). cpnDB: a chaperonin sequence database. *Genome Res.* 14 1669–1675. 10.1101/gr.2649204 15289485 PMC509277

[B24] Hossain M. T. Kim E.-Y. Kim Y.-R. Kim D.-G. Kong K. (2013). Development of a groEL gene-based species-specific multiplex polymerase chain reaction assay for simultaneous detection of *Vibrio cholerae*, *Vibrio parahaemolyticus* and *Vibrio vulnificus*. *J. Appl. Microbiol.* 114 448–456. 10.1111/jam.12056 23121500

[B25] Hung W. W. Chen Y. H. Tseng S. P. Jao Y. T. Teng L. J. Hung W. C. (2019). Using groEL as the target for identification of *Enterococcus faecium* clades and 7 clinically relevant *Enterococcus* species. *J. Microbiol. Immunol. Infect.* 52 255–264. 10.1016/j.jmii.2018.10.008 30473144

[B26] Itoh Y. Kawamura Y. Kasai H. Shah M. M. Nhung P. H. Yamada M. (2006). dnaJ and gyrB gene sequence relationship among species and strains of genus *Streptococcus*. *Syst. Appl. Microbiol.* 29 368–374. 10.1016/j.syapm.2005.12.003 16487673

[B27] Jung Y. J. Miller D. P. Perpich J. D. Fitzsimonds Z. R. Shen D. Ohshima J. (2019). Porphyromonas gingivalis tyrosine phosphatase php1 promotes community development and pathogenicity. *MBio.* 10:e2004-19. 10.1128/mBio.02004-19 31551334 PMC6759763

[B28] Junick J. Blaut M. (2012). Quantification of human fecal *Bifidobacterium* species by use of quantitative real-time PCR analysis targeting the *groEL* gene. *Appl. Environ. Microbiol.* 78 2613–2622. 10.1128/AEM.07749-7711 22307308 PMC3318781

[B29] Kwok S. Kellogg D. McKinney N. Spasic D. Goda L. Levenson C. (1990). Effects of primer-template mismatches on the polymerase chain reaction: human immunodeficiency virus type 1 model studies. *Nucleic Acids Res.* 18 999–1005. 10.1093/nar/18.4.999 2179874 PMC330356

[B30] Lara-Villoslada F. Olivares M. Sierra S. Rodriguez J. M. Boza J. Xaus J. (2007). Beneficial effects of probiotic bacteria isolated from breast milk. *Br. J. Nutr.* 98(Suppl. 1), S96–S100. 10.1017/S0007114507832910 17922969

[B31] Leigh W. J. Zadoks R. N. Jaglarz A. Costa J. Z. Foster G. Thompson K. D. (2018). Evaluation of PCR primers targeting the *groEL* gene for the specific detection of *Streptococcus agalactiae* in the context of aquaculture. *J. Appl. Microbiol.* 125 666–674. 10.1111/jam.13925 29786935

[B32] Lourenco J. Watkins E. R. Obolski U. Peacock S. J. Morris C. Maiden M. C. J. (2017). Lineage structure of *Streptococcus pneumoniae* may be driven by immune selection on the groEL heat-shock protein. *Sci. Rep.* 7:9023. 10.1038/s41598-017-08990-z 28831154 PMC5567354

[B33] Naveen Kumar V. van der Linden M. Menon T. Nitsche-Schmitz D. P. (2014). Viridans and bovis group streptococci that cause infective endocarditis in two regions with contrasting epidemiology. *Int. J. Med. Microbiol.* 304 262–268. 10.1016/j.ijmm.2013.10.004 24220665

[B34] Ogier J. C. Pages S. Galan M. Barret M. Gaudriault S. (2019). *rpoB*, a promising marker for analyzing the diversity of bacterial communities by amplicon sequencing. *BMC Microbiol.* 19:171. 10.1186/s12866-019-1546-z 31357928 PMC6664775

[B35] Oz H. S. Puleo D. A. (2011). Animal models for periodontal disease. *J. Biomed. Biotechnol.* 2011:754857. 10.1155/2011/754857 21331345 PMC3038839

[B36] Park S.-N. Kook J.-K. (2013). Development of *Streptococcus sanguinis*-, *Streptococcus parasanguinis*-, and *Streptococcus gordonii*-PCR primers based on the nucleotide sequences of species-specific DNA probes screened by inverted dot blot hybridization. *Int. J. Oral Biol.* 38 43–49. 10.11620/ijob.2013.38.2.043

[B37] Pattarachai K. Li L. Murray P. R. Fischer S. H. (2005). Use of housekeeping gene sequencing for species identification of viridans streptococci. *Diagn. Microbiol. Infect. Dis.* 51 297–301. 10.1016/j.diagmicrobio.2004.12.001 15808322

[B38] Sakamoto M. Umeda M. Ishikawa I. Benno Y. (2000). Comparison of the oral bacterial flora in saliva from a healthy subject and two periodontitis patients by sequence analysis of 16S rDNA libraries. *Microbiol. Immunol.* 44 643–652. 10.1111/j.1348-0421.2000.tb02545.x 11021394

[B39] Segata N. Waldron L. Ballarini A. Narasimhan V. Jousson O. Huttenhower C. (2012). Metagenomic microbial community profiling using unique clade-specific marker genes. *Nat. Methods* 9 811–814. 10.1038/nmeth.2066 22688413 PMC3443552

[B40] Songjinda P. Nakayama J. Kuroki Y. Tanaka S. Fukuda S. Kiyohara C. (2005). Molecular monitoring of the developmental bacterial community in the gastrointestinal tract of Japanese infants. *Biosci. Biotechnol. Biochem.* 69 638–641. 10.1271/bbb.69.638 15784997

[B41] Teng L. J. Hsueh P. R. Tsai J. C. Chen P. W. Hsu J. C. Lai H. C. (2002). *groESL* sequence determination, phylogenetic analysis, and species differentiation for viridans group *Streptococci*. *J. Clin. Microbiol.* 40 3172–3178. 10.1128/jcm.40.9.3172-3178.2002 12202549 PMC130726

[B42] Van den Bogert B. Boekhorst J. Herrmann R. Smid E. J. Zoetendal E. G. Kleerebezem M. (2013a). Comparative genomics analysis of *Streptococcus* isolates from the human small intestine reveals their adaptation to a highly dynamic ecosystem. *PLoS One* 8:e83418. 10.1371/journal.pone.0083418 24386196 PMC3875467

[B43] van den Bogert B. Erkus O. Boekhorst J. de Goffau M. Smid E. J. Zoetendal E. G. (2013b). Diversity of human small intestinal *Streptococcus* and *Veillonella* populations. *FEMS Microbiol. Ecol.* 85 376–388. 10.1111/1574-6941.12127 23614882

[B44] van den Bogert B. Meijerink M. Zoetendal E. G. Wells J. M. Kleerebezem M. (2014). Immunomodulatory properties of *Streptococcus* and *Veillonella* isolates from the human small intestine microbiota. *PLoS One* 9:e114277. 10.1371/journal.pone.0114277 25479553 PMC4257559

[B45] Vancuren S. J. Hill J. E. (2019). Update on cpnDB: a reference database of chaperonin sequences. *Database* 2019:baz033. 10.1093/database/baz033 30820575 PMC6395794

[B46] Viale A. M. Arakaki A. K. Soncini F. C. Ferreyra R. G. (1994). Evolutionary relationships among eubacterial groups as inferred from *GroEL* (chaperonin) sequence comparisons. *Int. J. Syst. Evol. Microbiol.* 44 527–533. 10.1099/00207713-44-3-527 7520741

[B47] Wang X. Lu H. Feng Z. Cao J. Fang C. Xu X. (2017). Development of human breast milk microbiota-associated mice as a method to identify breast milk bacteria capable of colonizing gut. *Front. Microbiol.* 8:1242. 10.3389/fmicb.2017.01242 28744259 PMC5504100

[B48] Watanabe K. Nelson J. Harayama S. Kasai H. (2001). ICB database: the gyrB database for identification and classification of bacteria. *Nucleic Acids Res.* 29 344–345. 10.1093/nar/29.1.344 11125132 PMC29849

[B49] Zhang C. Yin A. Li H. Wang R. Wu G. Shen J. (2015). Dietary modulation of gut microbiota contributes to alleviation of both genetic and simple obesity in children. *EBioMedicine* 2 968–984. 10.1016/j.ebiom.2015.07.007 26425705 PMC4563136

[B50] Zhou Q. Qin X. Qin M. Ge L. (2011). Genotypic diversity of *Streptococcus mutans* and *Streptococcus sobrinus* in 3-4-year-old children with severe caries or without caries. *Int. J. Paediatr. Dent.* 21 422–431. 10.1111/j.1365-263X.2011.01145.x 21689176

