# Supplementary material for: Quantification of Human Oral and Fecal Streptococcus parasanguinis by Use of Quantitative Real-Time PCR Targeting the groEL Gene
Source: Front Microbiol. 2019 Dec 20;10:2910. doi: 10.3389/fmicb.2019.02910 (PMC6933288; doi:10.3389/fmicb.2019.02910)
Supplement: Supplementary file 1 [file Data_Sheet_1.docx]

***Supplementary Material***

**Quantification of Human Oral and Fecal** ***Streptococcus parasanguinis* by Use of** **Quantitative Real-Time PCR Targeting the *groEL* Gene**

Qiurong Chen^1,2^, Guojun Wu^2^ , Hui Chen^2^ , Hui Li^2^, Shuo Li^2^, Chenhong Zhang^2^, Xiaoyan Pang^2^, Linghua Wang^2^, Liping Zhao^1,2^, Jian Shen^1 *^

^1^ Key Laboratory of Systems Biomedicine (Ministry of Education), Shanghai Center for Systems Biomedicine, Shanghai Jiao Tong University, Shanghai, China

^2^ State Key Laboratory of Microbial Metabolism, School of Life Sciences and Biotechnology, Shanghai Jiao Tong University, Shanghai, PR China

*** Correspondence:**Jian Shen
[shenjian@sjtu.edu.cn](mailto:shenjian@sjtu.edu.cn)

**Supplementary Tables**

# TABLE S1 The alignment of each of the unique sequences of degenerate primer Spa146f with the *groEL* gene of *S. parasanguinis* strains*^a^*

| Sequence origin*^b^* | Strain | GenBank accession No.  or Genome ID | Spa146f-1（5’ - 3’）  AACAATGCGATCCCAGTATCGAG | Spa146f-2（5’ - 3’）  AACAATGCGATCCCAGTATCAAG | Spa146f-3（5’ - 3’）AACAATGCGATTCCAGTATCGAG | Spa146f-4（5’ - 3’）  AACAATGCGATTCCAGTATCAAG |
| --- | --- | --- | --- | --- | --- | --- |
| The Chaperonin Sequence Database | ATCC 15912 | AF352799 | • • • • • • • • • • • • • • • • • • • • • • • |  |  |  |
|  | ATCC 903 | AEVE01000060 | • • • • • • • • • • • • • • • • • • • • • • • |  |  |  |
|  | FW213 | NC_017905 | • • • • • • • • • • • • • • • • • • • • • • • |  |  |  |
|  | M44 | GQ251514 | • • • • • • • • • • • • • • • • • • • • • • • |  |  |  |
|  | M688 | GQ251493 | • • • • • • • • • • • • • • • • • • • • • • • |  |  |  |
|  | F0405 | AEKM01000012 |  |  |  | • • • • • • • • • • • • • • • • • • • • • • • |
|  | SK236 | AFUC01000015 |  |  | • • • • • • • • • • • • • • • • • • • • • • • |  |
| NCBI genome collection database | BVME8 | GCA_001588805.1 | • • • • • • • • • • • • • • • • • • • • • • • |  |  |  |
|  | 889_SPAR | GCA_001074855.1 | • • • • • • • • • • • • • • • • • • • • • • • |  |  |  |
|  | 886_SPAR | GCA_001074805.1 | • • • • • • • • • • • • • • • • • • • • • • • |  |  |  |
|  | 65_SPAR | GCA_001073735.1 | • • • • • • • • • • • • • • • • • • • • • • • |  |  |  |
|  | 349_SPAR | GCA_001072435.1 | • • • • • • • • • • • • • • • • • • • • • • • |  |  |  |
|  | 392_SPAR | GCA_001071035.1 | • • • • • • • • • • • • • • • • • • • • • • • |  |  |  |
|  | 139.rep1_SPAR | GCA_001070575.1 | • • • • • • • • • • • • • • • • • • • • • • • |  |  |  |
|  | 139.rep2_SPAR | GCA_001069785.1 | • • • • • • • • • • • • • • • • • • • • • • • |  |  |  |
|  | FW213 | GCA_000262145.1 | • • • • • • • • • • • • • • • • • • • • • • • |  |  |  |
|  | F0449 | GCA_000260695.1 | • • • • • • • • • • • • • • • • • • • • • • • |  |  |  |
|  | ATCC 903 | GCA_000187505.1 | • • • • • • • • • • • • • • • • • • • • • • • |  |  |  |
|  | ATCC 15912 | GCA_000164675.2 | • • • • • • • • • • • • • • • • • • • • • • • |  |  |  |
|  | 348_SPAR | GCA_001072395.1 | • • • • • • • • • • • • • • • • • • • • • • • |  |  |  |
|  | VT517 | GCA_000963285.1 | • • • • • • • • • • • • • • • • • • • • • • • |  |  |  |
|  | DORA_23_24 | GCA_000508565.1 | • • • • • • • • • • • • • • • • • • • • • • • |  |  |  |
|  | DD19 | GCA_001578955.1 |  | • • • • • • • • • • • • • • • • • • • • • • • |  |  |
|  | MGH413 | GCA_000963275.1 |  | • • • • • • • • • • • • • • • • • • • • • • • |  |  |
|  | CC87K | GCA_000507765.1 |  | • • • • • • • • • • • • • • • • • • • • • • • |  |  |
|  | 540.rep2_SPAR | GCA_001076955.1 |  |  | • • • • • • • • • • • • • • • • • • • • • • • |  |
|  | 766_SPAR | GCA_001074295.1 |  |  | • • • • • • • • • • • • • • • • • • • • • • • |  |
|  | 318_SPAR | GCA_001072295.1 |  |  | • • • • • • • • • • • • • • • • • • • • • • • |  |
|  | 540.rep1_SPAR | GCA_001071295.1 |  |  | • • • • • • • • • • • • • • • • • • • • • • • |  |
|  | 451_SPAR | GCA_001071155.1 |  |  | • • • • • • • • • • • • • • • • • • • • • • • |  |
|  | 344_SPAR | GCA_001070915.1 |  |  | • • • • • • • • • • • • • • • • • • • • • • • |  |
|  | 1287_SPAR | GCA_001070445.1 |  |  | • • • • • • • • • • • • • • • • • • • • • • • |  |
|  | SK236 | GCA_000222725.2 |  |  | • • • • • • • • • • • • • • • • • • • • • • • |  |
|  | 512_SPAR | GCA_001073155.1 |  |  |  | • • • • • • • • • • • • • • • • • • • • • • • |
|  | F0405 | GCA_000180035.1 |  |  |  | • • • • • • • • • • • • • • • • • • • • • • • |
|  | C1A | GCA_000724645.1 |  |  | • • • • • • • • A • • • • • • • • • • • • • • |  |
|  | POW10 | GCA_001588725.1 |  |  | • • • • • • • • A • • • • • • • • • • • • • • |  |

*^a^*For each of the unique sequences of the degenerate primer, only the best alignment with individual reference sequences of known *S. parasanguinis* strains are shown. Degenerate primer Spa146f contains 4 unique sequences. • , Each dot represent one base of the primer which matches the corresponding base in the reference sequence of bacteria strain.

*^b^*The Chaperonin Sequence Database (<http://www.cpndb.ca>), NCBI genome collection database (<https://www.ncbi.nlm.nih.gov/>).

# TABLE S2 The alignment of each of the unique sequences of degenerate primer Spa93f with the *groEL* gene of *S. parasanguinis* strains*^a^*

| Sequence origin*^b^* | Strains | GeneBank accession no.  or Genome ID | Spa93f-1（5’ - 3’）  TCCGCCGTGGGATTGAGACC | Spa93f-2（5’ - 3’）  TCCGTCGTGGGATTGAGACC |
| --- | --- | --- | --- | --- |
| The Chaperonin Sequence Database | ATCC 15912 | AF352799 | • • • • • • • • • • • • • • • • • • • • |  |
|  | ATCC 903 | AEVE01000060 | • • • • • • • • • • • • • • • • • • • • |  |
|  | FW213 | NC_017905 | • • • • • • • • • • • • • • • • • • • • |  |
|  | M44 | GQ251514 | • • • • • • • • • • • • • • • • • • • • |  |
|  | M688 | GQ251493 | • • • • • • • • • • • • • • • • • • • • |  |
|  | F0405 | AEKM01000012 |  | • • • • • • • • • • • • • • • • • • • • |
|  | SK236 | AFUC01000015 |  | • • • • • • • • • • • • • • • • • • • • |
| NCBI genome collection database | DD19 | GCA_001578955.1 | • • • • • • • • • • • • • • • • • • • • |  |
|  | 889_SPAR | GCA_001074855.1 | • • • • • • • • • • • • • • • • • • • • |  |
|  | 766_SPAR | GCA_001074295.1 | • • • • • • • • • • • • • • • • • • • • |  |
|  | 349_SPAR | GCA_001072435.1 | • • • • • • • • • • • • • • • • • • • • |  |
|  | 451_SPAR | GCA_001071155.1 | • • • • • • • • • • • • • • • • • • • • |  |
|  | 344_SPAR | GCA_001070915.1 | • • • • • • • • • • • • • • • • • • • • |  |
|  | 139.rep1_SPAR | GCA_001070575.1 | • • • • • • • • • • • • • • • • • • • • |  |
|  | 139.rep2_SPAR | GCA_001069785.1 | • • • • • • • • • • • • • • • • • • • • |  |
|  | CC87K | GCA_000507765.1 | • • • • • • • • • • • • • • • • • • • • |  |
|  | FW213 | GCA_000262145.1 | • • • • • • • • • • • • • • • • • • • • |  |
|  | ATCC 903 | GCA_000187505.1 | • • • • • • • • • • • • • • • • • • • • |  |
|  | ATCC 15912 | GCA_000164675.2 | • • • • • • • • • • • • • • • • • • • • |  |
|  | 348_SPAR | GCA_001072395.1 | • • • • • • • • • • • • • • • • • • • • |  |
|  | VT517 | GCA_000963285.1 | • • • • • • • • • • • • • • • • • • • • |  |
|  | BVME8 | GCA_001588805.1 |  | • • • • • • • • • • • • • • • • • • • • |
|  | F0449 | GCA_000260695.1 |  | • • • • • • • • • • • • • • • • • • • • |
|  | SK236 | GCA_000222725.2 |  | • • • • • • • • • • • • • • • • • • • • |
|  | F0405 | GCA_000180035.1 |  | • • • • • • • • • • • • • • • • • • • • |
|  | 540.rep2_SPAR | GCA_001076955.1 |  | • • • • • • • • • • • • • • • • • • • A |
|  | 65_SPAR | GCA_001073735.1 |  | • • • • • • • • • • • • • • • • • • • T |
|  | 318_SPAR | GCA_001072295.1 |  | • • • • • • • • • • • • • • • • • • • A |
|  | 540.rep1_SPAR | GCA_001071295.1 |  | • • • • • • • • • • • • • • • • • • • A |
|  | 1287_SPAR | GCA_001070445.1 |  | • • • • • • • • • • • • • • • • • • • A |
|  | MGH413 | GCA_000963275.1 |  | • • • • • • • • • • • • • • • • • • • T |
|  | 886_SPAR | GCA_001074805.1 |  | • T • • • • • • • • • • • • • • • • • • |
|  | 392_SPAR | GCA_001071035.1 |  | • A • • • • • • • • • • • • • • • • • • |
|  | 512_SPAR | GCA_001073155.1 |  | • • • • • • • • • • • • • C • • • • • A |
|  | C1A | GCA_000724645.1 | • • • • • • • • • • • • • C • • • • • T |  |
|  | POW10 | GCA_001588725.1 | • • • • • • • • • • • • • C • • • • • • |  |
|  | DORA_23_24 | GCA_000508565.1 | TGATGG • • • • • • • GATGTAA |  |

*^a^*For each of the unique sequences of the degenerate primer, only the best alignment with individual reference sequences of known *S. parasanguinis* strains are shown. Degenerate primer Spa93f contains 2 unique sequences. • , Each dot represent one base of the primer which matches the corresponding base in the reference sequence of bacteria strain.

*^b^*The Chaperonin Sequence Database (<http://www.cpndb.ca>), NCBI genome collection database (<https://www.ncbi.nlm.nih.gov/>).

# TABLE S3 The alignment of each of the unique sequences of degenerate primer Spa525r with the *groEL* gene of *S. parasanguinis* strains*^a^*

| Sequence origin*^b^* | Strains | GeneBank accession no. or Genome ID | Spa525r-1  CTACGACATTAAAGGTACCGCGG | Spa525r-2  CTACGACATTAAAGGTACCACGG | Spa525r-3  CTACGACATTAAAGGTACCTCGG |
| --- | --- | --- | --- | --- | --- |
| The Chaperonin Sequence Database | ATCC 15912 | AF352799 | • • • • • • • • • • • • • • • • • • • • • • • |  |  |
|  | ATCC 903 | AEVE01000060 |  | • • • • • • • • • • • • • • • • • • • • • • • |  |
|  | FW213 | NC_017905 | • • • • • • • • • • • • • • • • • • • • • • • |  |  |
|  | M44 | GQ251514 | • • • • • • • • • • • • • • • • • • • • • • • |  |  |
|  | M688 | GQ251493 | • • • • • • • • • • • • • • • • • • • • • • • |  |  |
|  | F0405 | AEKM01000012 | • • • • • • • • • • • • • • • • • • • • • • • |  |  |
|  | SK236 | AFUC01000015 |  |  | • • • • • • • • • • • • • • • • • • • • • • • |
| NCBI genome collection database | BVME8 | GCA_001588805.1 | • • • • • • • • • • • • • • • • • • • • • • • |  |  |
|  | DD19 | GCA_001578955.1 | • • • • • • • • • • • • • • • • • • • • • • • |  |  |
|  | 889_SPAR | GCA_001074855.1 | • • • • • • • • • • • • • • • • • • • • • • • |  |  |
|  | 886_SPAR | GCA_001074805.1 | • • • • • • • • • • • • • • • • • • • • • • • |  |  |
|  | 766_SPAR | GCA_001074295.1 | • • • • • • • • • • • • • • • • • • • • • • • |  |  |
|  | 65_SPAR | GCA_001073735.1 | • • • • • • • • • • • • • • • • • • • • • • • |  |  |
|  | 512_SPAR | GCA_001073155.1 | • • • • • • • • • • • • • • • • • • • • • • • |  |  |
|  | 349_SPAR | GCA_001072435.1 | • • • • • • • • • • • • • • • • • • • • • • • |  |  |
|  | 318_SPAR | GCA_001072295.1 | • • • • • • • • • • • • • • • • • • • • • • • |  |  |
|  | 451_SPAR | GCA_001071155.1 | • • • • • • • • • • • • • • • • • • • • • • • |  |  |
|  | 392_SPAR | GCA_001071035.1 | • • • • • • • • • • • • • • • • • • • • • • • |  |  |
|  | 344_SPAR | GCA_001070915.1 | • • • • • • • • • • • • • • • • • • • • • • • |  |  |
|  | 139.rep1_SPAR | GCA_001070575.1 | • • • • • • • • • • • • • • • • • • • • • • • |  |  |
|  | 1287_SPAR | GCA_001070445.1 | • • • • • • • • • • • • • • • • • • • • • • • |  |  |
|  | 139.rep2_SPAR | GCA_001069785.1 | • • • • • • • • • • • • • • • • • • • • • • • |  |  |
|  | MGH413 | GCA_000963275.1 | • • • • • • • • • • • • • • • • • • • • • • • |  |  |
|  | FW213 | GCA_000262145.1 | • • • • • • • • • • • • • • • • • • • • • • • |  |  |
|  | F0449 | GCA_000260695.1 | • • • • • • • • • • • • • • • • • • • • • • • |  |  |
|  | F0405 | GCA_000180035.1 | • • • • • • • • • • • • • • • • • • • • • • • |  |  |
|  | ATCC 15912 | GCA_000164675.2 | • • • • • • • • • • • • • • • • • • • • • • • |  |  |
|  | 348_SPAR | GCA_001072395.1 | • • • • • • • • • • • • • • • • • • • • • • • |  |  |
|  | VT517 | GCA_000963285.1 | • • • • • • • • • • • • • • • • • • • • • • • |  |  |
|  | 540.rep2_SPAR | GCA_001076955.1 |  | • • • • • • • • • • • • • • • • • • • • • • • |  |
|  | 540.rep1_SPAR | GCA_001071295.1 |  | • • • • • • • • • • • • • • • • • • • • • • • |  |
|  | CC87K | GCA_000507765.1 |  | • • • • • • • • • • • • • • • • • • • • • • • |  |
|  | ATCC 903 | GCA_000187505.1 |  | • • • • • • • • • • • • • • • • • • • • • • • |  |
|  | SK236 | GCA_000222725.2 |  |  | • • • • • • • • • • • • • • • • • • • • • • • |
|  | POW10 | GCA_001588725.1 |  | • • • • A • • • • • • • • • • • • • • • • • • |  |
|  | C1A | GCA_000724645.1 |  | • • • • A • • • • • • • • • • • • • • • • • • |  |
|  | DORA_23_24 | GCA_000508565.1 |  | • • • • A • • • • • • • • • • • • • • • • • • |  |

*^a^*For each of the unique sequences of the degenerate primer, only the best alignment with individual reference sequences of known *S. parasanguinis* strains are shown. Degenerate primer Spa525r contains 3 unique sequences. • , Each dot represent one base of the primer which matches the corresponding base in the reference sequence of bacteria strain.

*^b^*The Chaperonin Sequence Database (<http://www.cpndb.ca>), NCBI genome collection database (<https://www.ncbi.nlm.nih.gov/>).

# TABLE S4 The bacterial strains of which *groEL* gene can be amplified with the designed primer pairs as predicted *in silico* by the Simulated PCR (SPCR) algorithm under different product amplification coefficients*^a^*

| Product amplification  coefficient *^b^* | Spa146f / Spa525r | Spa93f / Spa525r | Phylogeny *^c^* |
| --- | --- | --- | --- |
| 0.8 | ***S. parasanguinis* ATCC 15912 (b1123)** | ***S. parasanguinis* ATCC 15912 (b1123)** | ***S. parasanguinis*** |
|  | ***S. parasanguinis* M44 (b16689)** | ***S. parasanguinis* M44 (b16689)** | ***S. parasanguinis*** |
|  | ***S. parasanguinis* M688 (b16710)** | ***S. parasanguinis* M688 (b16710)** | ***S. parasanguinis*** |
|  | ***S. parasanguinis* ATCC 15912 (b17957)** | ***S. parasanguinis* ATCC 15912 (b17957)** | ***S. parasanguinis*** |
|  | ***S. parasanguinis* F0405 (b18125)** | ***S. parasanguinis* F0405 (b18125)** | ***S. parasanguinis*** |
|  | ***S. parasanguinis* ATCC 903 (b18513)** | ***S. parasanguinis* ATCC 903 (b18513)** | ***S. parasanguinis*** |
|  | ***S. parasanguinis* SK236 (b19467)** | ***S. parasanguinis* SK236 (b19467)** | ***S. parasanguinis*** |
|  | ***S. parasanguinis* FW213 (b21140)** | ***S. parasanguinis* FW213 (b21140)** | ***S. parasanguinis*** |
|  | ***Streptococcus. sp*. C520 (b16687)** | **Streptococcus. sp. C520 (b16687)** | ***S. parasanguinis*** |
|  | ***Streptococcus*. sp. F0442 (b21707)** | ***Streptococcus*. sp. F0442 (b21707)** | ***S. parasanguinis*** |
|  |  | *S. lutetiensis* N9 N9 (b15950 | *S. lutetiensis* |
|  |  | *S. lutetiensis* ATCC BAA-103 (b7991) | *S. lutetiensis* |
|  |  | *S. infantarius* N1 N1 (b16004) | *S. infantarius* |
|  |  | *Streptococcus. sp.* W8 W8 G5-1 (b15945) | *S. infantarius* |
|  |  | *Streptococcus. sp.* z1227 (b357) | *S. infantarius* |
| 0.9 | ***S. parasanguinis* ATCC 15912 (b1123)** | ***S. parasanguinis* ATCC 15912 (b1123)** | ***S. parasanguinis*** |
|  | ***S. parasanguinis* M44 (b16689)** | ***S. parasanguinis* M44 (b16689)** | ***S. parasanguinis*** |
|  | ***S. parasanguinis* M688 (b16710)** | ***S. parasanguinis* M688 (b16710)** | ***S. parasanguinis*** |
|  | ***S. parasanguinis* ATCC 15912 (b17957)** | ***S. parasanguinis* ATCC 15912 (b17957)** | ***S. parasanguinis*** |
|  | ***S. parasanguinis* F0405 (b18125)** | ***S. parasanguinis* F0405 (b18125)** | ***S. parasanguinis*** |
|  | ***S. parasanguinis* ATCC 903 (b18513)** | ***S. parasanguinis* ATCC 903 (b18513)** | ***S. parasanguinis*** |
|  | ***S. parasanguinis* SK236 (b19467)** | ***S. parasanguinis* SK236 (b19467)** | ***S. parasanguinis*** |
|  | ***S. parasanguinis* FW213 (b21140)** | ***S. parasanguinis* FW213 (b21140)** | ***S. parasanguinis*** |
|  | ***Streptococcus. sp.* C520 (b16687)** | ***Streptococcus. sp.* C520 (b16687)** | ***S. parasanguinis*** |
|  | ***Streptococcus. sp.* F0442 (b21707)** | ***Streptococcus. sp.* F0442 (b21707)** | ***S. parasanguinis*** |

*^a^*866 *groEL* universal target (UT) sequences of 64 Streptococcus spp., which included 8 sequences of 7 *S. parasanguinis* strains, were downloaded from the Chaperonin Sequence Database (<http://www.cpndb.ca>), and introduced into SPCR as templates. The SPCR algorithm output the template sequences that can be amplified by the tested primer pair under each coefficient. The accession numbers of the *groEL* sequences in GenBank are shown in parentheses following the bacterial names.

*^b^*Increase of the product amplification coefficient corresponds to the enhancement of the annealing temperature in experimental PCR. 0.80 is the recommended value according to the SPCR developer.

*^c^*The phylogeny of the *groEL* gene predicted to produce amplicons with either primer pair was determined by the phylogenetic tree in supplementary figure S1

# TABLE S5 The abundance of *S. parasanguinis* in human feces determined by metagenomic sequencing, qPCR with primer pair Spa93f-Spa525r, and qPCR with Spa146f-Spa525r

| Fecal Sample ID | metagenomic sequencing (%) *^a^* | qPCR with Spa146f/ Spa525r (copies/ng DNA) | qPCR with Spa93f/Spa525r (copies/ng DNA) |
| --- | --- | --- | --- |
| PWS01 | 3.07409 | 33443 | 10191 |
| PWS02 | 0.44554 | 978 | 290 |
| PWS03 | 0.34287 | 532 | 56 |
| PWS04 | 0.36788 | 631 | 231 |
| PWS05 | 0.20942 | 356 | 73 |
| PWS06 | 0.09817 | 422 | 80 |
| PWS07 | 0.12723 | 311 | 78 |
| PWS08 | 0.17447 | 952 | 265 |
| PWS09 | 0.13034 | 967 | 148 |
| PWS10 | 0.06825 | 148 | 26 |
| PWS11 | 0.05044 | 219 | 38 |
| PWS12 | 0.05328 | 50 | 9 |
| PWS13 | 0.04813 | 130 | 33 |
| PWS14 | 0.03097 | 156 | 23 |
| PWS15 | 0.03071 | 162 | 72 |
| PWS16 | 0.01823 | 142 | 54 |
| PWS17 | 0.01641 | 237 | 24 |
| PWS18 | 0.00863 | 84 | 11 |
| PWS19 | 0.00204 | 44 | 10 |
| PWS20 | 0 | 0 | 0 |
| PWS21 | 0 | 0 | 0 |
| PWS22 | 0 | 0 | 0 |

*^a^*Data were derived from the metagenomic sequencing in our previous study([Zhang et al., 2015](#_ENREF_2)) . The metagenomic relative abundances of *S. parasanguinis* in human feces were calculated using MetaPhlAn([Segata et al., 2012](#_ENREF_1)).

# **TABLE S6** The abundances of saliva *S. parasanguinis* in periodontitis patients and orally healthy people determined by qPCR with primer pair Spa93f-Spa525r and Spa146f-Spa525r

| Volunteer group | Sample ID | Spa146f and Spa525r (copies/ng DNA) | Spa93f and Spa525r (copies/ng DNA) |
| --- | --- | --- | --- |
| Patients | SP1 | 15 | 0 |
|  | SP2 | 99750 | 32363 |
|  | SP3 | 27070 | 11512 |
|  | SP4 | 449 | 206 |
|  | SP5 | 56540 | 28483 |
|  | SP6 | 25950 | 11323 |
|  | SP7 | 42 | 0 |
|  | SP8 | 718 | 422 |
|  | SP9 | 77870 | 9209 |
|  | SP10 | 12270 | 4890 |
|  | SP11 | 1519000 | 1024367 |
|  | SP12 | 27900 | 4609 |
|  | SP13 | 2105000 | 1101800 |
|  | SP14 | 45500 | 27270 |
|  | SP15 | 99140 | 56660 |
|  | SP16 | 9019 | 2124 |
|  | SP17 | 568 | 1059 |
|  | SP18 | 2477 | 0 |
|  | SP19 | 16800 | 2077 |
|  | SP20 | 139 | 0 |
|  | SP21 | 69850 | 17950 |
|  | SP22 | 30540 | 14577 |
|  | SP23 | 8041 | 366 |
|  | SP24 | 265300 | 171100 |
|  | SP25 | 6668 | 534 |
|  | SP26 | 17210 | 1063 |
|  | SP27 | 46050 | 24313 |
|  | SP28 | 4240 | 0 |
| Healthy | SH1 | 3154 | 284 |
|  | SH2 | 3873 | 988 |
|  | SH3 | 65360 | 36413 |
|  | SH4 | 44410 | 21300 |
|  | SH5 | 85250 | 18893 |
|  | SH6 | 44680 | 10340 |
|  | SH7 | 7708 | 4619 |
|  | SH8 | 341900 | 188733 |
|  | SH9 | 222700 | 112367 |
|  | SH10 | 41560 | 11017 |
|  | SH11 | 126200 | 47540 |
|  | SH12 | 245300 | 153933 |
|  | SH13 | 29810 | 11680 |
|  | SH14 | 249800 | 20545 |
|  | SH15 | 688800 | 585033 |
|  | SH16 | 3920 | 483 |
|  | SH17 | 106600 | 42087 |
|  | SH18 | 281800 | 88290 |
|  | SH19 | 35950 | 11442 |
|  | SH20 | 51030 | 6676 |
|  | SH21 | 35950 | 8929 |
|  | SH22 | 17210 | 8395 |
|  | SH23 | 1215 | 591 |
|  | SH24 | 160700 | 99257 |
|  | SH25 | 830600 | 557567 |
|  | SH26 | 268500 | 136700 |

**Supplementary Figures**


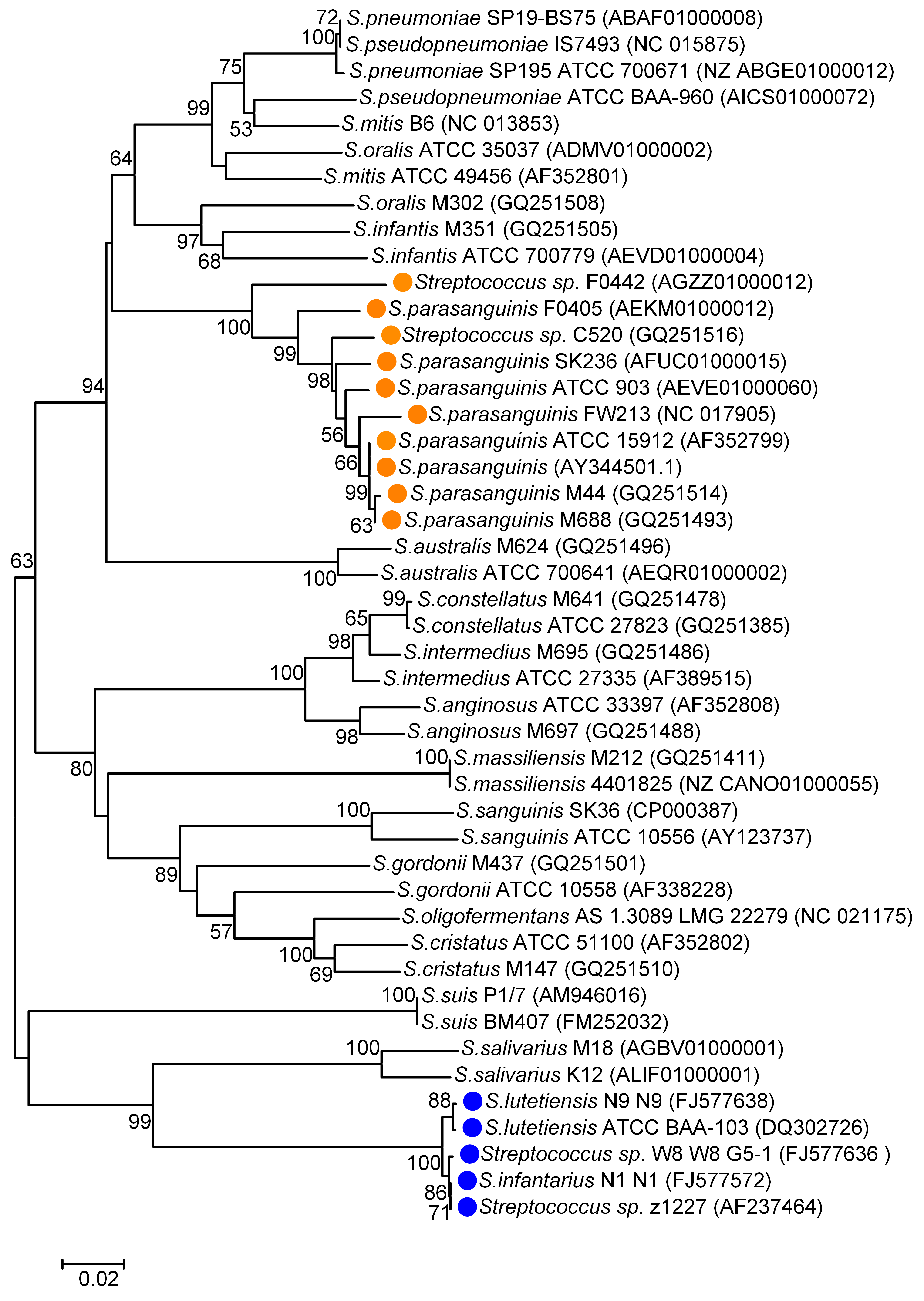


**Supplementary Figure S1** Phylogenetic tree of *groEL* universal target (UT) sequences predicted to produce amplicons with either primer pair according to SPCR algorithm and those of known *Streptococcus* species. The *groEL* universal target (UT) sequences predicted to produce amplicons with either primer pair are labeled by dots, and orange dots are *S. parasanguinis* strains, and blue dots are non- *S. parasanguinis* bacteria. The accession numbers of the *groEL* genes are given in parentheses following the bacterial names. The phylogenetic robustness was assessed by bootstrap analysis with 1000 replicates, and bootstrap values greater than 50% are indicated at the nodes.


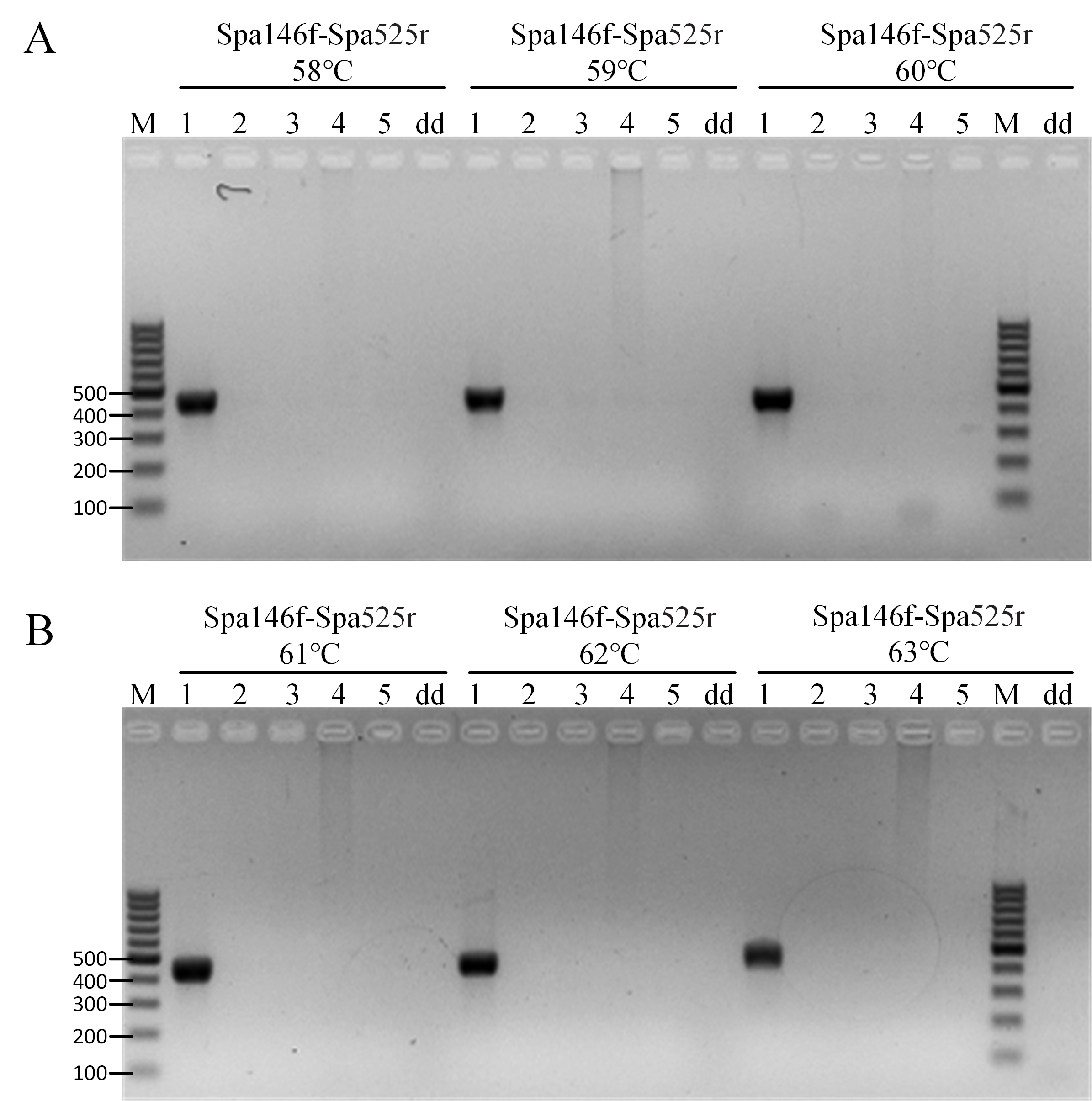


**Supplementary Figure S2** The agarose gel electrophoresis of the PCR amplicons produced with primer pair Spa146f/Spa525r using genomic DNA of different *Streptococcus* species as templates under gradient annealing temperatures. 1, *S. parasanguinis*; 2, S*. salivarius*; 3, *S. sanguinis* ATCC 10556; 4, *S. mutans* UA159; 5, *S. gordonii* ATCC 10558; dd, Negative control in which template DNA was replaced by sterilized distilled water; M, DNA marker


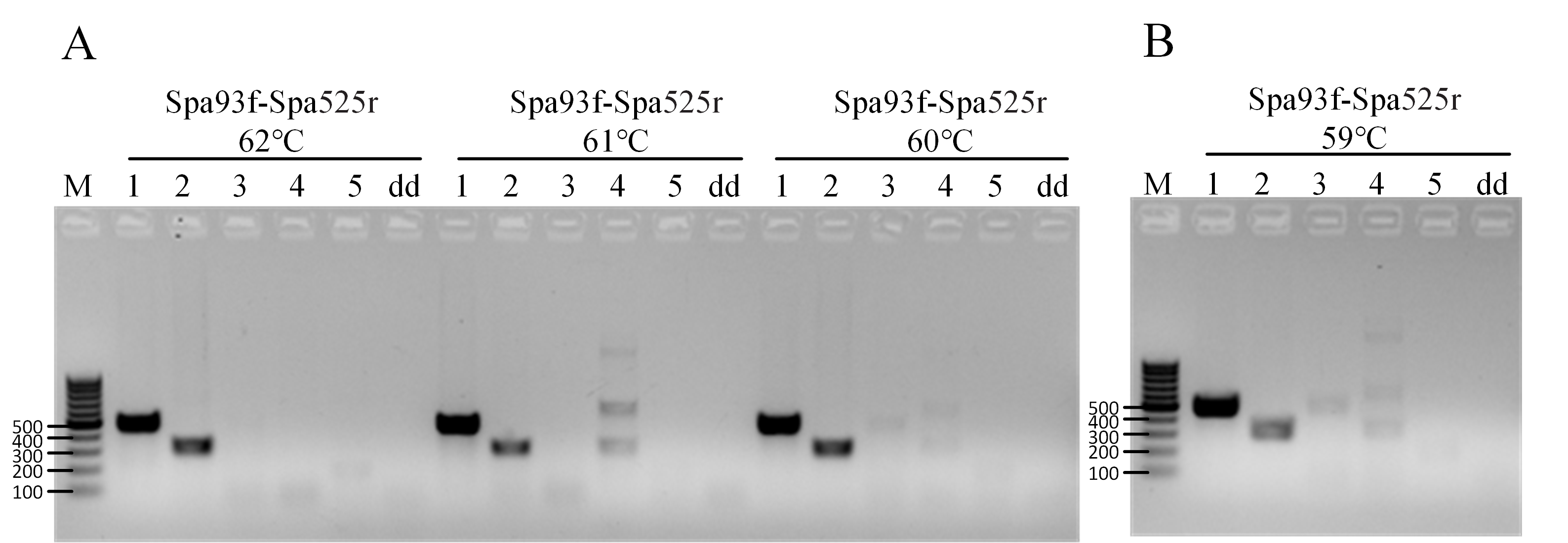


**Supplementary Figure S3** The agarose gel electrophoresis of the PCR amplicons produced with primer pair Spa93f/Spa525r using the genomic DNA of different *Streptococcus* species as templates under gradient annealing temperatures. 1, *S. parasanguinis*; 2, S*. salivarius*; 3, *S. sanguinis* ATCC 10556; 4, *S. mutans* UA159; 5, *S. gordonii* ATCC 10558; dd, Negative control in which template DNA was replaced by sterilized distilled water; M, DNA marker


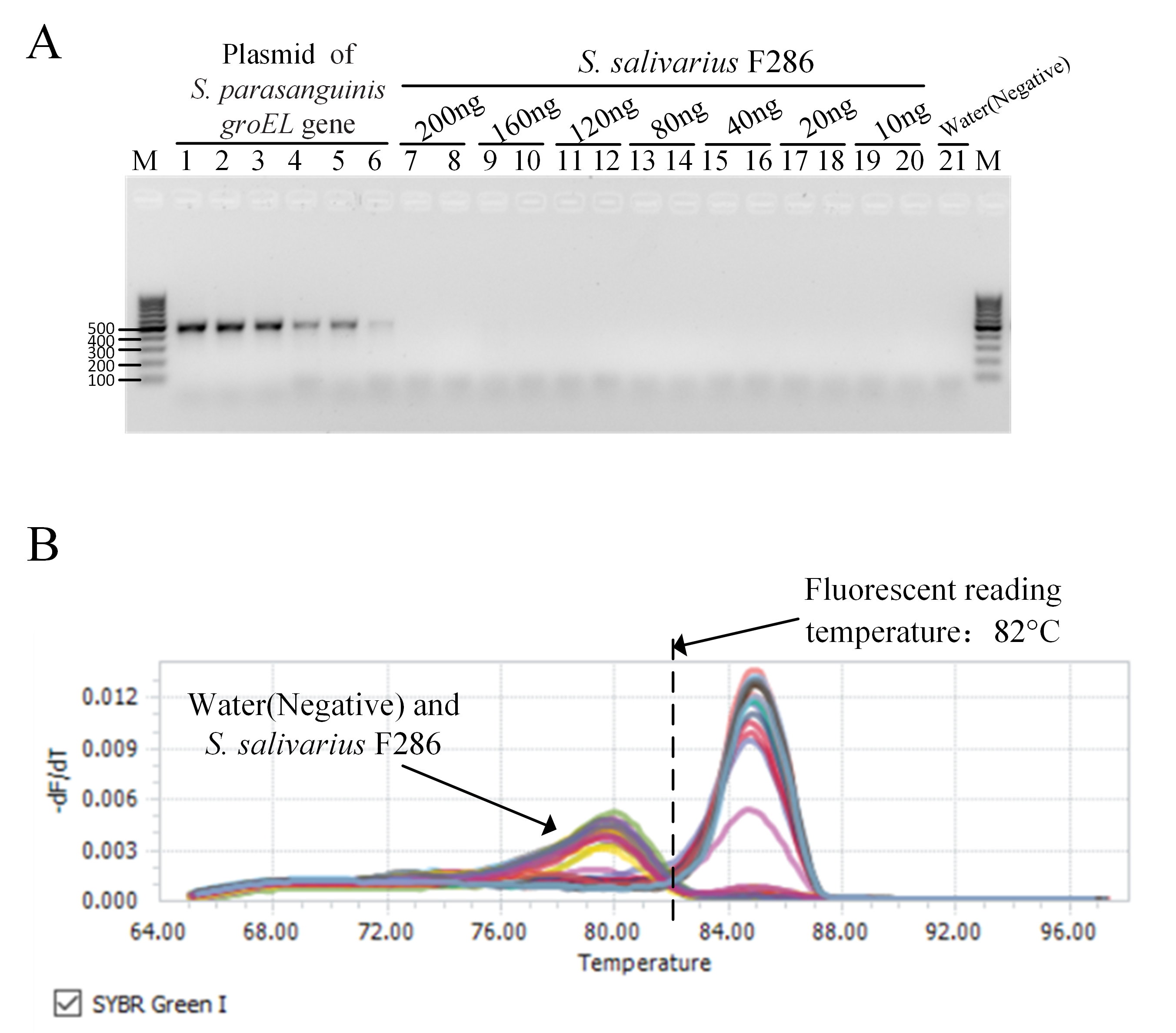


**Supplementary Figure S4** Spa93f-Spa525r did not produce amplicons with *S. salivarius* genomic DNA as template in qPCR assays. Serving as the positive control, the plasmids containing the *S. parasanguinis* *groEL* gene at different concentrations were used as templates for amplification in qPCR assays. As negative control, sterile distilled water was added to the qPCR mixture as template. (A) The 1.5% agarose gel of qPCR amplicon generated with primer Spa93f /Spa525r using S*. salivarius* genomic DNA of different amounts as templates. 3 μl PCR products were loaded on the agarose gel. (B) The melting curves of qPCR amplicons generated with Spa93f /Spa525r using S*. salivarius* genomic DNA of different concentrations as templates. The water negative controls and assays templating *S. salivarius* genomic DNA showed peaks that were probably primer dimers according to the agarose gel of the qPCR products shown in (A).


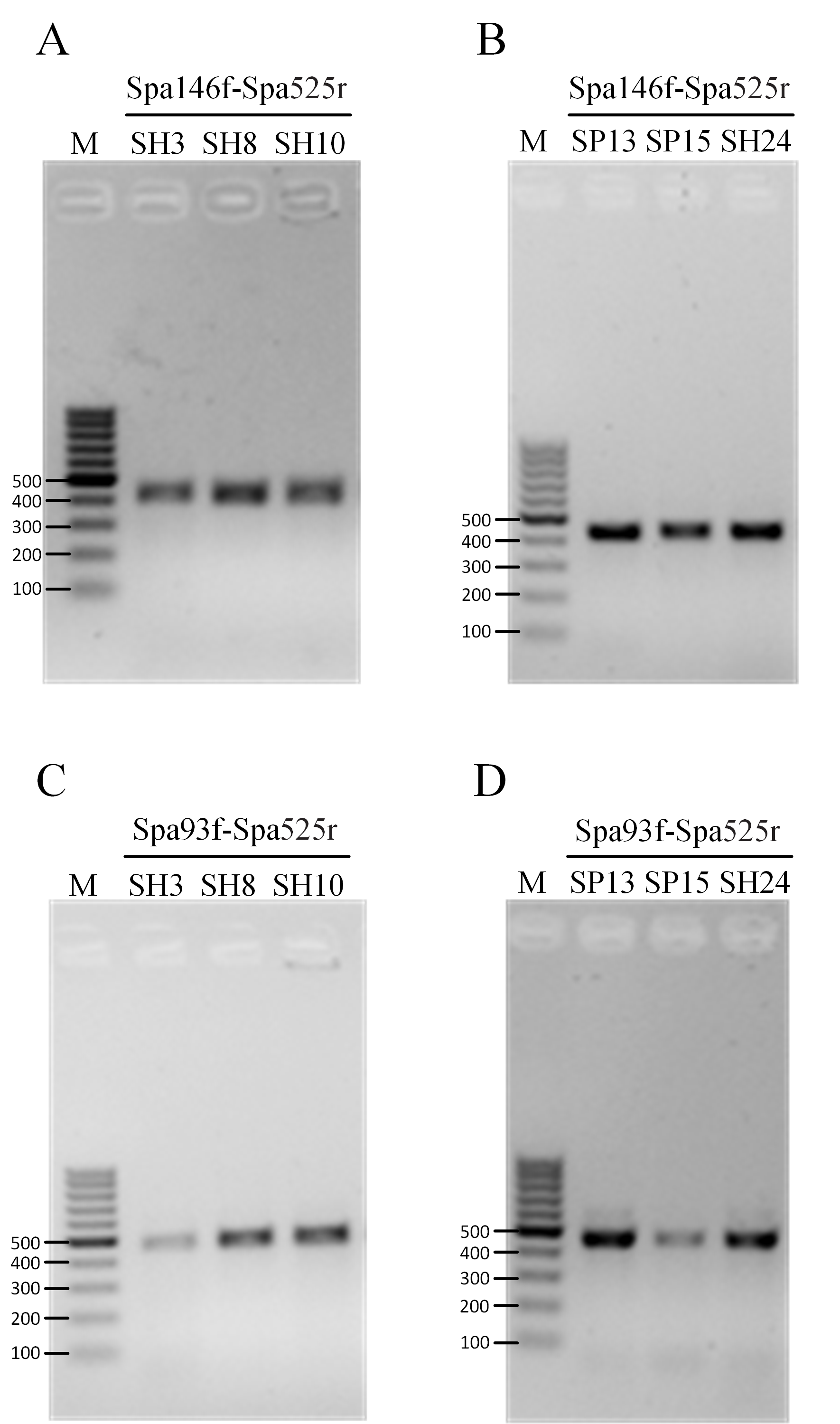


**Supplementary Figure S5** The agarose gel electrophoresis of the PCR amplicons produced with primer pair Spa146f/Spa525r and Spa3f/Spa525r, respectively, using saliva DNA of 6 human subjects as templates. Spa146f-Spa525r and Spa93f-Spa525r produced specific amplicons of expected sizes with human saliva DNA as templates. SH3, SH8, SH10, SP13, SP15, and SP24 are the IDs of the human subjects. M, DNA marker.





**Supplementary Figure S6** Phylogenetic tree of the *groEL* gene fragments cloned by the primer pair Spa146f-Spa525r from the saliva of 6 human subjects. The cloned sequences are labeled by dots of varied colors, and clones with the identical color are from the saliva sample of one person. Bootstrap values greater than 50% are indicated at the nodes. (A) Tree of sequences cloned with primer pair Spa146f-Spa525r. (B) Tree of sequences cloned with primer pair Spa93f-Spa525r.

#


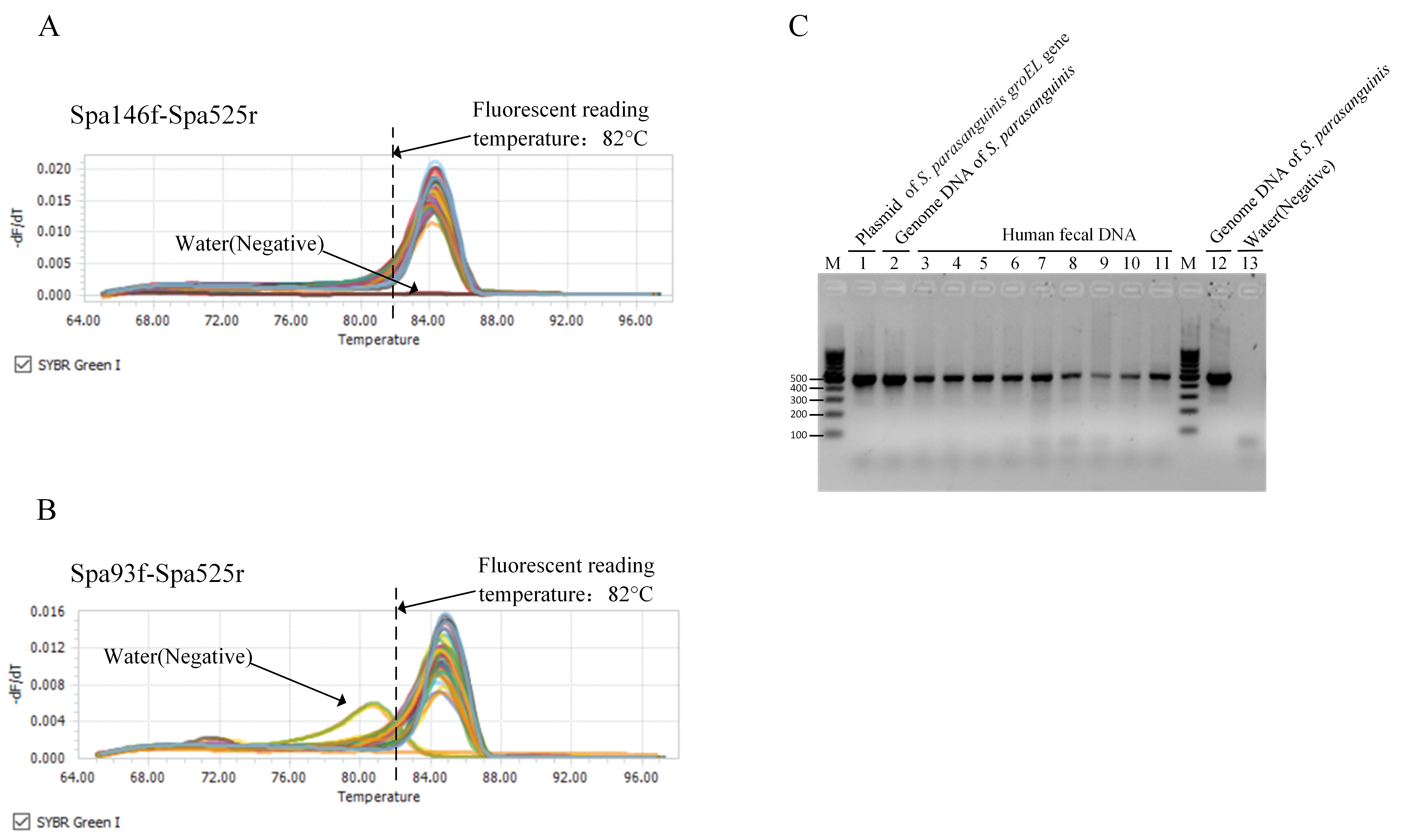


**Supplementary Figure S7** Spa146f-Spa525r and Spa93f-Spa525r did not produce non-specific amplicons in human fecal qPCR assays. As negative control, sterile distilled water was added to the qPCR mixture as template. (A) The melting curves of fecal qPCR amplicons produced with primer pair Spa146f-Spa525r. (B) The melting curves of fecal qPCR amplicons produced with primer pair Spa93f-Spa525r. The water negative controls showed a peak that was probably primer dimers according to the agarose gel of the qPCR products shown in (C). (C) The 1.5% agarose gel of fecal qPCR amplicons generated with primer Spa93f /Spa525r. Serving as the positive controls, qPCR amplicons of assays using the genomic DNA of *S. parasanguinis* F278 strain and the plasmids containing the *S. parasanguinis groEL* gene as the templates were loaded. 3 μl PCR products were loaded on the agarose gel.


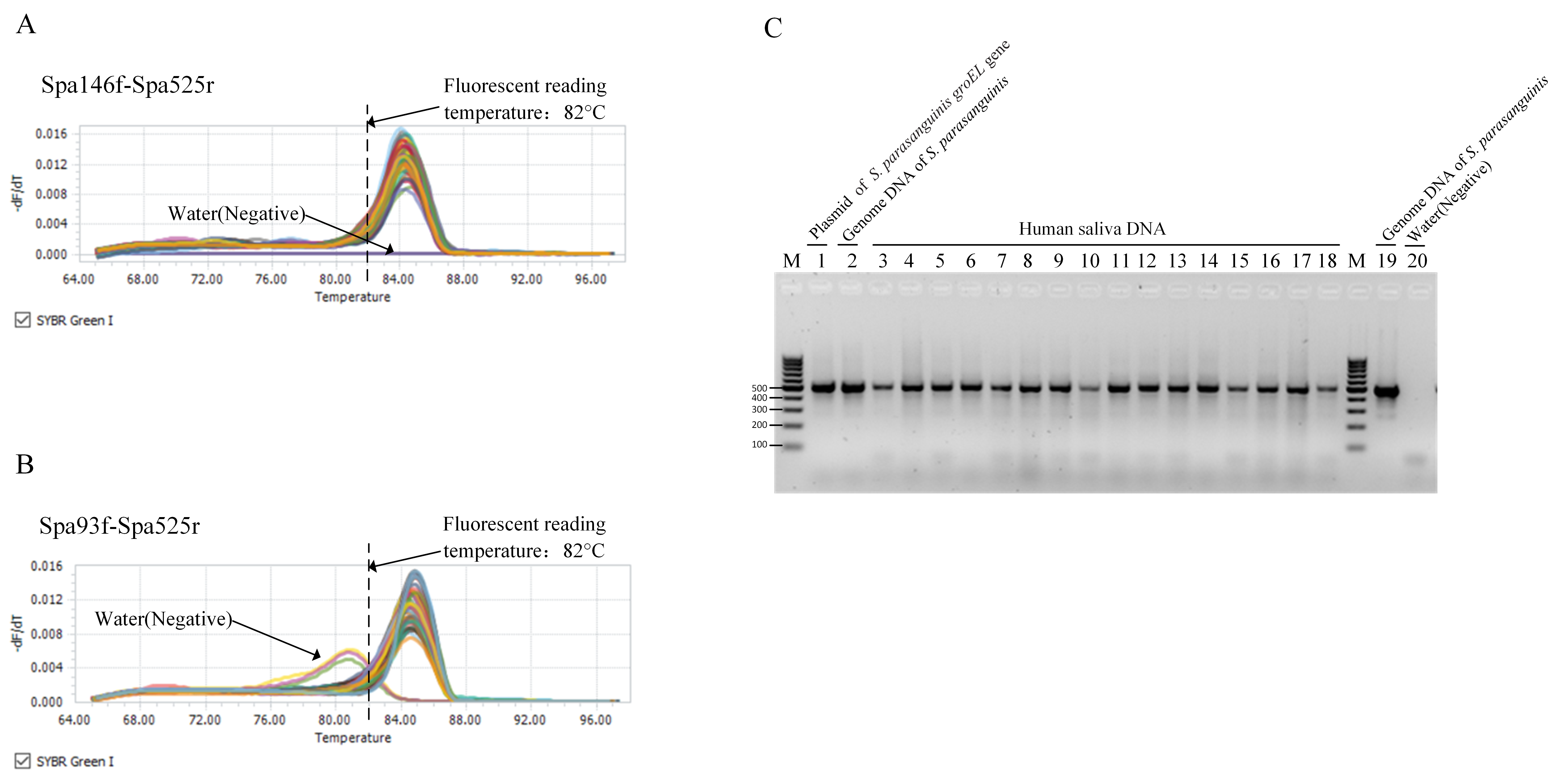


**Supplementary Figure S8** Spa146f-Spa525r and Spa93f-Spa525r did not produce non-specific amplicons in human saliva qPCR assays. As negative control, sterile distilled water was added to the qPCR mixture as template. (A) The melting curves of saliva qPCR amplicons produced with primer pair Spa146f-Spa525r. (B) The melting curves of saliva qPCR amplicons produced with primer pair Spa93f-Spa525r. The water negative controls showed a peak that was probably primer dimers according to the agarose gel of the qPCR products shown in (C). (C) The 1.5% agarose gel of saliva qPCR amplicons generated with primer Spa93f /Spa525r. Serving as the positive controls, qPCR amplicons of assays using the genomic DNA of *S. parasanguinis* F278 strain and the plasmids containing the *S. parasanguinis groEL* gene as the templates were loaded. 3 μl PCR products were loaded on the agarose gel.

**References**

Segata, N., Waldron, L., Ballarini, A., Narasimhan, V., Jousson, O., and Huttenhower, C. (2012). Metagenomic microbial community profiling using unique clade-specific marker genes. *Nat Methods* 9(8)**,** 811-814. doi: 10.1038/nmeth.2066.

Zhang, C., Yin, A., Li, H., Wang, R., Wu, G., Shen, J., et al. (2015). Dietary Modulation of Gut Microbiota Contributes to Alleviation of Both Genetic and Simple Obesity in Children. *EBioMedicine* 2(8)**,** 968-984. doi: 10.1016/j.ebiom.2015.07.007.
